# Drug-Eluting Stents and Balloons—Materials, Structure Designs, and Coating Techniques: A Review

**DOI:** 10.3390/molecules25204624

**Published:** 2020-10-11

**Authors:** I. Rykowska, I. Nowak, R. Nowak

**Affiliations:** 1 Faculty of Chemistry, Adam Mickiewicz University, Uniwersytetu Poznańskiego 8, 61-614 Poznań, Poland; grzesiw@amu.edu.pl; 2 Eye Department, J. Strus City Hospital, Szwajcarska 3, 61-285 Poznań, Poland; raf.nowak@wp.pl

**Keywords:** controlled drug delivery, drug-eluting stents, drug-eluting balloons, polymers

## Abstract

Controlled drug delivery is a matter of interest to numerous scientists from various domains, as well as an essential issue for society as a whole. In the treatment of many diseases, it is crucial to control the dosing of a drug for a long time and thus maintain its optimal concentration in the tissue. Heart diseases are particularly important in this aspect. One such disease is an obstructive arterial disease affecting millions of people around the world. In recent years, stents and balloon catheters have reached a significant position in the treatment of this condition. Balloon catheters are also successfully used to manage tear ducts, paranasal sinuses, or salivary glands disorders. Modern technology is continually striving to improve the results of previous generations of stents and balloon catheters by refining their design, structure, and constituent materials. These advances result in the development of both successive models of drug-eluting stents (DES) and drug-eluting balloons (DEB). This paper presents milestones in the development of DES and DEB, which are a significant option in the treatment of coronary artery diseases. This report reviews the works related to achievements in construction designs and materials, as well as preparation technologies, of DES and DEB. Special attention was paid to the polymeric biodegradable materials used in the production of the above-mentioned devices. Information was also collected on the various methods of producing drug release coatings and their effectiveness in releasing the active substance.

## 1. Introduction

The constrained supply of medicines is one of the hottest topics dealt with in modern pharmacology. Monitoring the kinetics of drug release has many benefits for patients’ health. It provides an improvement in the effectiveness of the drug-based treatment and reduces the severity of side effects. The sustainable drug release systems allow the active substance to be delivered precisely to the affected place in the right dose.

The concept of controlled drug delivery systems (CDDS) refers to ensuring “programmed” drug releases over time and delivering the drugs to a specific place in the body. Research in the field of CDDS is being conducted in terms of:

- the mechanisms of releasing the active substances (e.g., by diffusion, ion exchange, or osmosis);
- release kinetics;
- materials used as carriers and delivery routes;
- medicines that can be used for appropriate therapy.

Meeting all of the CDDS objectives (appropriate drug concentration, delivery time, and delivery target place) is a considerable challenge.

For decades, cardiovascular diseases have been at the forefront of death statistics. One of the most commonly used treatments for coronary heart disease [1] is percutaneous transluminal coronary angioplasty (PTCA) with stents and balloon catheters. The idea of a cardiovascular stent has revolutionized coronary disease-related therapies [2,3,4]. In turn, balloon angioplasty (BA), first introduced in 1977 by Grüntzig, was a further step in the development of treatment techniques for coronary heart diseases [5]. Later advancements in this field included the first human implantation of a self-expanding stent in 1986 [6], and further in 1987 [7], the first human implantation of a balloon-expandable stent [8].

There are many types of endovascular prostheses and many criteria for their classification. The most commonly used categorization is the division into classic metal stents (BMS—bare metal stents) and drug-eluting stents (DES—drug-eluting stents or DCS—drug-coated stents) [9].

The first bare-metal stents (BMS) were quickly supplemented with modern systems supplemented by drug-eluting coatings. Releasing pharmaceutical agents from the stent surface was promising progress in the realm of cardiovascular stents [2]. However, it soon turned out that there are some limitations in delivering the sufficient amount of drugs in a suitable time frame by DES. The first DES released only small amount of the drug and only in one portion, which, in most cases, proved insufficient [10].

It is assumed that the drug release process will be slow and programmed for controlled delivery [11]. Moreover, drugs for DES should have a selective mode of action targeted at preventing in-stent restenosis, including (1) the capability of inhibiting the platelet aggregation, inflammation, smooth muscle cell (SMC) proliferation, and migration and (2) promoting appropriate healing and fast endothelialization [12].

Despite the supreme advantages of DESs over BMSs, in-stent restenosis (ISR) and long-term safety remain ongoing concerns in the clinical application of DESs. The prospective success of DES for long-term clinical applications depends on the following areas:

- permanent polymeric coating materials;
- metallic stent platforms;
- optimal drug-releasing condition, and
- the factors that have recently been identified as disadvantages, such as:
  ○ the degradation of the products of polymers and
  ○ the presence of metal ions due to the erosion and degradation of metals and their alloys utilized in some stents as a metal base [13].

Determining the direct relationship between stent materials and possible adverse effects is very difficult and has still not been done. For clinical success, it is essential to design DES that overcomes the problems commonly present in in-stent transplantology, such as the inflammatory response, delayed endothelialisation, and subacute stent thrombosis (ST) [2], simultaneously.

Drug-eluting balloons (DEB) introduced in recent years can be such an alternative in some clinical situations [14]. The motivation for using DEB is based on the limitations of DES, such as the risk of developing late thrombosis, caused by the incomplete endothelialization of the stent, damage to the endothelial function, and inflammatory response of the vessel to the presence of a polymer. In addition to the risk of thrombosis, the risk of restenosis cannot be ignored.

Despite the existence of many types of stents and balloon catheters on the market, there is still a need to look for new technologies and materials to ensure their better mechanical, physicochemical, and biomedical properties.

The number of publications on DES is increasing every year, reflecting the intensification of research related to this domain.

This work is a literature review of the latest developments in the field of drug-releasing stents and balloons. The text collects a wide range of information and the latest scientific achievements related to these devices. It describes, among others, the compositions of stents and balloons, the drug-release mechanism, and coating techniques. The paper refers to the most frequently used DES preparation procedures, such as coating, immersion, and spraying methods. Attention was paid to the low quality of the above-mentioned mechanical coating techniques, and studies showing new ways of stent loading improving the quality of DES were cited [15,16].

Since polymers are an essential component of DES and DEB and, at the same time, remain the most controversial part of the technology of these devices, this work collected the information on the most commonly used and currently studied polymers [17]. It reports the debates and solutions in this field.

## 2. Cardiovascular Stent Design Parameters

Stent design parameters may be listed as follows: the dimension of the stent struts, the full expansion of the stent, the radial strength of the stent, the extent of the balloon injury during the stent deployment, the nature of the disease itself (the intensity of the obstruction of the artery), the ability to tolerate the compression exerted by the vessel wall, the minimum longitudinal contraction by the time of expanding, and the amount of flexibility of the stent, especially for curved vessels to suitably flex in them [3,18,19].

The stent material are required to be nonerodible, noncytotoxic, resorbable, flexible, radioopaque, biocompatible, compatible with the chemical nature of the drug, and ideally, to have sufficient radial strength.

Titanium (Ti) and its alloys have been reported as potential materials for the stent backbone, with excellent biocompatibility and corrosion resistance as a result of a stable oxide layer on the surface. A new Ti-based alloy was proposed by Saleh et al. [13] as the stent platform with decorated nanostructures on the surface. So far, there are limited reports on the application of Ti and its alloys for the stent material.

In turn, the selection of the covering material for the stent surface must be made according to the following criteria. The surface coating must be appropriate for the best adhesion of drugs, be compatible with drug molecules, and be biocompatible [3].

In general, materials to be implanted or injected in the human body need to be both chemically and mechanically stable in the biological environment for long-term use. Thus, deciding for a potential biomaterial, a dual approach must be undertaken [2]: (1) studying the biodegradation of the material in the host tissue environment, as well as the safety of biodegradation products to impede sensitivity in the local site of the prosthesis, and second, (2) studying the behavior of the biomaterial during its presence in the body [4].

## 3. Drug-Eluting Stents (DES)

Monitoring of the drug release process in the physiological environment is a crucial aspect of an effective treatment technique. A time-programmed drug release can make a balance between the drug release to the artery lumen and the drug absorption by the surrounding artery tissue. The key is to choose the right system for delivering medicine. The rapid rate of drug release might exceed the tissue uptake. In contrast, a slow release rate could delay the tissue-healing process [20]. Contemporary coronary stent technology continues to improve the performance of previous-generation devices by enhancing their design, structure, and component materials. These technologies include:

- new generations of drug-eluting stents,
- nonpolymeric stents,
- bioresorbable polymer-coated stents, and
- fully bioresorbable scaffolds [1].

Table 1 gathers a review of publications presenting and analyzing the latest developments in these devices.

**Figure 1:**
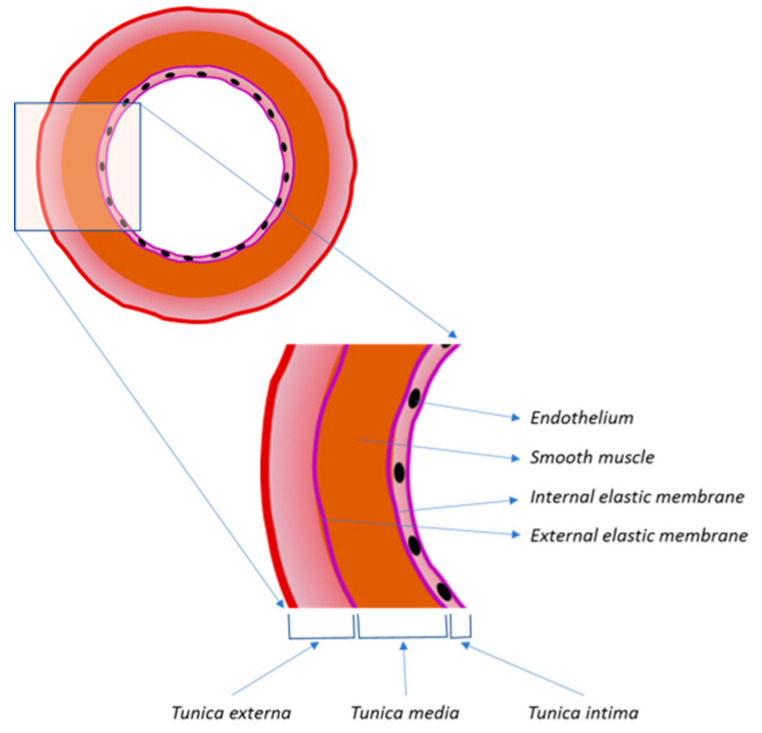
The structure on an artery wall. Three layers form the standard artery wall: the tunica intima, the tunica media, and the tunica adventitia (externa). The intimia (the most inner one) consists of the endothelium (a single layer of cells), connective tissue, and a basal layer of elastic tissue called the internal elastic lamina. Concentric layers of vascular smooth muscle cells and elastin-rich extracellular matrix make the tunica media, which is separated from adventitia by the external elastic lamina. The tunica adventitia is the outer layer and is formed by fibroblasts, collagen, mast cells, nerve endings, and vasa vasorum [35].

Stents can be ideal drug delivery systems, because they allow local releasing of the active ingredient into the vascular damaged area. Besides, the drug released from the stent can have the capability to inhibit the complex cascade of events that lead to building a new tunica intima after stent implantation (Figure 1). The potential objectives of therapeutic interventions are to limit the proliferation of the new intimia, to define inflammatory and proliferative mechanisms associated with the healing process, and to induce vascular repair processes specific to the blood and vascular wall.

Prime DESs consisted of three main parts: a permanent metallic platform, a durable polymeric coating, and an active pharmaceutical agent incorporated into the polymeric surface that was being eluted from the polymeric layer [36]. Additionally, the first DES were made not only of steel (iron) but also of nickel, which, according to some authors, could cause recurrent allergic stenosis (3). Nonetheless, these devices have outperformed BMSs in reducing neointimal proliferation and restenosis based on clinical studies [37].

### 3.1. Polymer-Coated Stents (PCS)

Significant developments in the field of vascular prostheses have resulted in the use of matrix polymer coatings to release medicinal substances. Unlike classical metal stents, polymer-coated stents (PCS) show high plasticity and facilitate the placement of the prosthesis at the implantation site. The polymeric coating is, therefore, a suitable matrix for the controlled immobilization and release of antiproliferative drugs. Polymer layers on the stent surface have the following roles: (1) inhibiting the drug from being washed off from the stent surface, (2) providing a suitable scaffold for drug loading, (3) providing an engineered control over the drug release, and (4) providing a satisfactory platform for biocompatibility [3]. The polymer-based top coating layer was employed for inhibiting any burst release of drugs to have a longer drug elution to the site of action [3]. Unfortunately, polymer-coated stents show some mechanical limitations related to stent coating damage, such as cracks, flaking, and delamination. Besides, despite the undeniable advantages leading to a sustainable release of drugs, there is also a risk of the long-term persistence of nondegradable polymers at the site of vessel damage, which may lead to late stent thrombosis (ST) [36].

Based on the findings, the requirements for PCS have been established as follows [15]. Firstly, the flexibility of the PCS should allow stretching without delamination or disintegration of the stent [38]. Secondly, such a polymer should be selected to enable the pharmaceuticals placed in its structure to be released at a constant, controlled, and predictable rate [39]. Thirdly, the implant polymer should be stable, biocompatible with vascular tissue, chemically compatible with drugs, and able to control the release of drugs [40]. Additionally, it is essential that the polymer coating retain the mechanical integrity of the DES during its implantation. It is also crucial to have a comprehensive knowledge of the stent components and their possible interactions with the host tissues. It determines the safety and effectiveness of DES [2].

First-generation DES does not match today’s medical standards [36]. Serious clinical events cast doubt on the efficiency of DES in terms of its long-term safety because of the increased risk of late and very late ST. Thus, potential technical problems associated with the use of DES include:

- delayed endothelialization caused by the locally delivered drugs,
- inherent thrombogenicity of the stent as a foreign body to the immune system,
- hypersensitivity and inflammatory reactions due to the base framework and/or polymeric coatings,
- insufficient drug amount in addition to a lack of sustained drug release, and
- stent displacement [13].

### 3.2. Biodegradable Materials for DES

Since it was recognized that nonbiodegradable polymers could initiate an inflammatory response contributing to in-stent restenosis (ISR), polymeric materials with increased biocompatibility and biodegradability were proposed for stent skeleton construction. These much safer stents with thinner struts are known as second-generation DES [41].

Biodegradable polymers have also been proposed as a coating material in DES to avoid adverse pathological effects and better control of drug elution [37]. Biodegradable DES-coating polymers became a revolutionary solution [2] and opened the way for charging stents with different types of active substances.

#### 3.2.1. Bioresorbable Materials for Scaffolds

The idea of a new generation of stents based on biodegradable scaffolds, which is still undergoing R&D, was a big step in stent technology. This solution can give temporary support to the artery and fully biodegrades after completing its functionality. Scaffolds, as stated in papers [39,42,43], play a crucial role in the vascular restoration therapy associated with endothelial function and vasomotion.

Bioresorbable cardiovascular scaffolds (BRS) are a promising alternative to permanent stents in cardiology [44]. The term scaffold indicates the temporary nature of BRS, which is in opposition to a permanent implant [45]. Properties of an ideal biodegradable platform were identified as follows [44,46]:

- biocompatibility: before, during, and after degradation;
- adequate radial strength;
- the proper time of degradation: not too fast to increase inflammation and not too long to provoke adverse body reactions—usually 4–6 months;
- no inflammatory process initiative by degradation;
- compatibility with DES technology, eluting drugs at a determined rate without any effect on the radial strength;
- thin struts;
- easy deliverability;
- enhanced visualization under fluoroscopy;
- compatible with currently available equipment for deployment; and
- improved dwell time before implementation.

The absence of a permanent core has the potential to overcome the shortcomings of the conventional BMS or metal-based DES [44]. Biodegradable scaffolding is temporary, i.e., it undergoes bioresorption after exhaustion of the drug supply and completion of the recovery process. In the next stage, the stent disappears from the site through bioresorption. This type of stent allows the vessel to return to its initial natural state without any blockage [47]. Some highlights about BRS that make this device superior to BMS and metal-based DES are adaptive shear stress, late luminal gain, late expansive remodeling, reduction in restenosis and late-stent thrombosis, reintervention possibility at the site of injury, and improved invasive imaging. Besides, BRSs show a better capacity to restore natural vascular function and higher flexibility in comparison to metal backbones. All of these bright sides can represent a significant advance in interventional surgery for cardiovascular diseases [41].

Researchers indicate the following potential benefits of using biodegradable stents: (1) restoration of cyclic pulsatility and normal vasomotion, (2) preventing ST, (3) normalization of shear stress and cyclic strain, (4) preventing constrictive remodeling, (5) reducing the risk of very late polymer reactions, (6) reducing atherosclerosis, (7) avoidance of late-vessel wall inflammation, (8) unjailing of side branches, (9) preventing acute occlusion, (10) restoration of normal vessel curvature, (11) preventing severe recoil, (12) preventing extensive remodeling, (13) avoidance of stent malapposition, (14) avoidance of late-luminal enlargement, (15) avoiding neointimal hyperplasia, and (16) a formation of a cap over the lipid-rich plaque. There is also no shortage of opinions among scientists indicating potential risks associated with the use of biodegradable stents. These include (1) unsuitable release profile for a drug delivery system, (2) higher risk of acute strut fracture as a result of insufficient mechanical strength compared with metallic DES, (3) increased rates of early thrombosis, (4) specific (cold) storage conditions and (5) specific deployment techniques, (6) difficulty in delivery to the site of action because of thicker struts with more extensive crossing profiles, (7) inadequate degradation and resorption profiles, (8) and inflammatory degradation residues [47,48,49,50].

Biodegradable stents are promising candidates for vast future clinical applications. However, degradation of the stent is still the most concerning issue due to vessel recoil problems and hypersensitivity [4].

It is important to mention that, until recently, surgeons were more likely to choose metal-based stents (BMS over metal-based DES) rather than polymer-based ones (BRS), since the metal platform has a higher mechanical strength. Moreover, by loading stents with heparin (Hep), it was also possible to gain control of thrombosis [10]. It should be remembered, however, that metal stents have many disadvantages, which have already been mentioned in this paper.

Despite the predominance of polymers as a stent material, there are still uncertainties related to their use that require analysis: (1) understanding the mechanical behavior during and after implantation at the site of the injury and during its degradation and (2) comparing the efficacy of polymer-based and metal-based stents [41].

Among the potential complications arising from the use of polymers as a base material, it should be taken into account:

- lower stiffness and strength of polymer materials that make stent struts be thicker in comparison to conventional metal-based stents;
- increase in diameters of struts may lead to complications within the stent, such as platelet adhesion, and vessel injury;
- at a stress level below the yield and tensile strength of the material under consideration, premature destruction of a polymer may happen; the result is that, long before the polymer is degraded, the device fails in the face of the liquid pressure and the exerted pressure from the vessel wall; and
- mechanical behavior of the polymer and other bioresorbable polymers, due to molecular weight, temperature, molecular orientation, the crystallinity of the polymer, and degradation characteristics, is nonlinear.

Thus, it is of high importance to be familiar with a polymer’s behaviors before applying the device in the human body. Analyzing the stent action under simulated body conditions could help researchers make the best choice of a stent material and its composition [51]. Assessing the polymer features contributing to the stent function is possible through experimental analysis accompanied by analytical and numerical studies.

#### 3.2.2. Biodegradable Materials as Drug Release Coating Materials

Drug-releasing polymers have found many applications in medical devices and are the most useful materials for the treatment of coronary artery disease. Biodegradable polymers have been widely investigated for use in tissue engineering and drug delivery. Among the desired features of a polymer, carriers are stable, compatible with drugs, nonflammability, and have sterilization resistance. However, it should be noted that polymers remain the most controversial component of drug-eluting technology [17].

Table 2 presents selected review publications on polymers that apply to drug-eluting systems.

The polymers used for manufacturing implantable drug delivery devices may be divided into two categories: biodegradable and nonbiodegradable polymers [57]. The review of Zhao et al. discusses the last 15 years of research devoted to the efficacy of polymer stents based on (poly (l-lactic acid (PLLA)). It covered a wide range of studies involving the mechanical testing of PLLA materials and PLLA-based scaffolds and computational studies that have been opening a new perspective to the predictions of outcomes [41].

There are many studies based on natural and synthetic polymer materials used in stent technology [58]. Synthetic polymers are generally biologically inert, have predictable chemical and physical properties, and do not have the same batch-to-batch inconsistency that occurs with natural polymers [58,59]. The biodegradability and biocompatibility of any material are critical when designing a drug delivery system [60]. Any materials used must be fully biocompatible, and any changes in polymer properties that develop as it degrades must be thoroughly investigated and characterized [61]. Ideally, any chosen biodegradable polymer should be quickly metabolized and excreted by physiological pathways, degradable to nontoxic products, and free from an inflammatory response in vivo [61,62].

The first-generation drug-eluting stents were sirolimus (Cypher^®^) and paclitaxel (Taxus^®^) releasing stents. These stents were made of stainless steel scaffolding (SS) coated with nonbiodegradable polymers poly(n-butyl methyl acrylate) (PBMA) and poly(ethylene-co-vinyl acetate) (PEVA) or poly(styrene-b-isobutylene-b-styrene) (PSIBS). Their applications resulted in a remarkable reduction of the usually occurring restenosis [63]. However, safety concerns have been raised regarding the possibility of late-stent thrombosis in the case of its long-term use [64].

**Figure 2:**
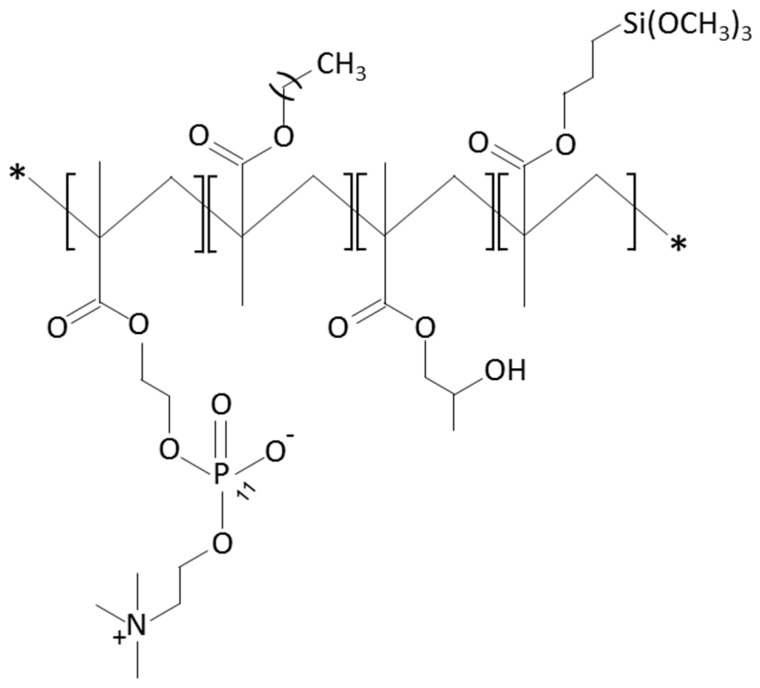
Schematic representation of the chemical structure of an exemplary phosphorylcholine polymer [56].

Over the next few years, the second generation of DES with thinner struts and incorporated with novel, more effective drugs were introduced. As in the first-generation DES, synthetic, nonbiodegradable polymers such as PBMA, PEVA, PSIBS, poly(hexafluoropropylene) (PHFP), and poly(vinylidene fluoride) (PVDF), in combination with phosphorylcholine polymer (PCh), were used (Figure 2) [65]. Table 3 presents the polymer-coated DES approved by the FDA [10,66,67,68,69,70] to date.

A serious disadvantage of the first- and second-generation stents is that the nonbiodegradable polymers used for their coatings remain on the stent after complete release of the drug, which may cause local hypersensitivity, tissue inflammation, and delayed vessel healing. Such stents may cause thrombosis in the late stage of DES [68].

Recently, third-generation stents covered with fully biodegradable polymers such as PLGA and PLA have appeared on the market. [71,72]. One example is the everolimus-eluting stent, which consists of a platinum-chromium (Pt-Cr) platform coated with a biodegradable PLGA copolymer. A summary of the FDA and Conformité Européenne (CE)-approved DES based on biodegradable polymers are listed in Table 4 [66,73,74,75,76,77].

The presented-above solutions increased the effectiveness and viability of stents, but the problem of the metal scaffold (BRS) remaining in the artery after biopolymer degradation stayed unresolved [37]. A significant disadvantage of nonbiodegradable implants is the potential possibility of polymer accumulation in the body, which requires their surgical removal [78]. Therefore, there is still a need to search for fully biodegradable stents [56] the body would efficiently excrete after the task completion [61].

The latest achievement in low-invasive CAD (Coronary Artery Disease) treatment is the creation of the fourth generation of DES, which has a biodegradable polymer core modified with an active substance.

The biodegradable polymers most often used as construction materials for scaffolds and as surface coatings for stents are described below.

##### Biodegradable Polymers

The thermoplastic aliphatic poly(esters), including poly(lactic acid) (PLA), poly(glycolic acid) (PGA), and poly(lactic-co-glycolic acid) (PLGA), have been widely examined due to their favorable characteristics such as biodegradability, biocompatibility, and mechanical strength [79,80,81]. Table 5 presents the chemical structures of PLA, PGA, PLGA, and PCL and mechanisms of their degradation. In Table 6, the mechanical and thermal properties of the mentioned medical biodegradable polymers are gathered. 

These polymers have been previously successfully used in nanoparticle-based drug delivery systems and solid and microparticle parenteral implants [80]. Degradation periods for these polymers range from one month to more than six months [82]. The degradation rate is affected by factors such as hydrophilicity, glass transition temperature T_g,_ and molecular weight and environmental conditions such as temperature and pH [59,81,82].

##### Poly(lactic acid)

Regarding degradable polymers, the PLLA (Poly (L-lactic acid) of different molecular weights is the base material of vascular and cardiovascular stents. Polylactic acid is a popular polymer for medical applications. It is the first polymer used to build a stent scaffold that, in 2016, got FDA approval for use in direct contact with biological fluids, as it is a generally recognized as safe (GRAS) material [83]. PLA is biodegradable and bioresorbable. It can be obtained by polymerizing lactic acid of natural origin (e.g., rice, maize, or potato starch) [83,84,85,86]. PLA shows similar mechanical properties to other synthetic polymers and is characterized by a relatively low production cost. Due to its semipermeability to oxygen and water, it is more susceptible to biodegradation compared to other biomedical synthetic polymers [84,85,87,88]. At room temperature, PLA is a white powder showing melting and glass transition temperatures of around 175 and 55 °C, respectively [86]. Due to the existence of two stereoisomers of lactic acid (D and L), PLA can be made using both types of monomers [86]. PLA prepared using D-lactic acid, PDLA, is a crystalline material due to its regular chain structure [86]. In turn, PLA made using L-lactic acid, PLLA, is a semicrystalline material [86]. The mixing of both these polymers gives, in turn, an amorphous polymer (PDLLA) [86]. All these polymers are soluble in a wide variety of organic solvents such as benzene, chloroform, acetonitrile, tetrahydrofuran, or dioxane [86]. Due to their hydrophobic nature, PLAs are insoluble in ethanol, methanol, and aliphatic hydrocarbons [86].

PLLA is a semicrystalline polymer with the random or amorphous segments, which are distributed throughout the polymer structure between the ordered polymer chains known as crystal lamella. Crystallinity brings out mechanical strength to the system and facilitates the dispersion of drug molecules in the polymer matrix. The amorphous segment determines the rate of degradation, while the crystal domain of the polymer determines the absorption rate [44,49]. It is known that the degree of crystallinity is vital for the degradation rate of the polymer. The crystalline domains within a polymer have a low affinity to water molecules, which brings a slower rate of degradation as a result of poor hydrolysis [89]. On the other hand, the amount of applied stress will also influence the rate of polymer degradation. The mechanical factors consisted of yield stress, yield strain, and elongation at break when the decrease was significantly conducive to polymer destruction.

The chirality of the monomer influences the biodegradability and mechanical properties of PLA. It has been established that D and D/L forms of PLA degrade more rapidly than the L form, as the latter has a higher crystallinity [84,90,91,92,93]. Increasing the surface area-to-volume ratio or the porosity of the polymer will improve the rate of degradation of the polymer [94]. The main PLA mechanism of degradation is the hydrolysis of the ester bond backbone [95].

PLA can be processed using a wide variety of techniques due to its high thermal processability [86]. Accordingly, it can be used in extrusion, film casting, blow molding, or fiber-spinning processes, among others [86]. It is an excellent advantage over other biomaterials, such as poly(ethylene glycol). PLA production requires between 25% and 55% less fossil energy than petroleum-based polymers [86]. For these reasons, PLA is the second-most traded polymer in the world [85].

The products obtained in the degradation are lactic acid or lactic acid oligomers. Interestingly, the degradation is catalyzed by the newly formed terminal carboxylic acid groups at the ends of the PLA chains [96]. Temperature and pH influence the degradability of the material. PLA showed higher degradation rates at physiological temperatures higher than at 25 °C. Furthermore, at lower pH, the degradation of this polymer is much slower than at the physiological pH [97].

In addition to PLA hydrolysis, this polymer can be enzymatically biodegraded. After implantation of the polymer in the body, immune cells are directed to the implantation site. These cells secrete enzymes, including lactate dehydrogenase and acid phosphatase, that contribute to PLA degradation [98].

##### Poly(glycolic acid)

Poly(glycolic acid) (PGA) is a polyester made by the polymerization of glycolic acid units. It was one of the first biodegradable polymers used for biomedical applications. PGA is a polymer that exists in only one highly crystalline form [90,99]. It exhibits excellent mechanical properties (higher than those of PLA) and a melting point greater than 200 °C [58]. Biodegradable sutures made from PGA have been successfully used—for example, Dexon^®^ [58]. PGA exhibits a quick degradation profile, and it is insoluble in many common solvents. PGA undergoes bulk degradation via scission of its ester backbone to form glycine, which is excreted in the urine or via the citric acid cycle [58,90]. However, the acidic by-products of PGA can cause inflammation in the surrounding tissues [90] and limit the potential use of PGA as a lone polymer. Accordingly, this polymer has not been used alone for drug delivery purposes.

##### Poly(lactic-co-glycolic acid)

Poly(lactic-co-glycolic acid) (PLGA) is a biodegradable and biocompatible copolymer of PLA and PGA [95]. PLGA degrades in the body via hydrolysis to form lactic acid and glycolic acid [80,100].

PLGA, therefore, presents itself as an attractive candidate as a polymer for implantable drug delivery devices [82]. It is possible to modify the physical properties of the polymer by altering the polymer molecular weight and ratio of lactide to glycolide [82]. The presence of side methyl groups within PLA make the copolymer more hydrophobic. Thus, PLGA copolymers with high PLA contents show higher hydrophobicity and, consequently, slower degradation rates. The advantages of PLGA include an increased degradation rate in comparison to PLLA; however, it decreased in comparison to PDLA, and a lack of acidic by-products were produced upon degradation [90]. The monomer composition and the molecular weight of the PLGA molecules have a direct influence in the crystallinity of the polymer. Similar to the previously described polymers, the mechanical properties and the degradation rates are strongly influenced by the degree of crystallinity of the polymer. A higher PGA content within PLGA leads to a lower crystallinity degree and a higher rate of hydration/hydrolysis. PLGA containing 50:50 of PLA-PGA shows the highest degradation rates. PLGA copolymers present the glass transition temperature T_g_ (values above 37 °C); thus, exhibiting a fairly rigid chain structure, they are ideal for implant manufacturing.

##### Poly(caprolactone)

Poly(caprolactone) (PCL) is a perspective material for use in polymeric implants due to its biocompatibility, biodegradability, nontoxicity, and relatively low cost [58,101]. It has FDA approval for use in medical applications [102], and it has already been successfully incorporated into materials used for sutures and wound dressings [103]. The presence of unstable aliphatic ester bonds allows the polymer to biodegrade by a mixture of random hydrolysis of ester bonds and bulk degradation pathways [58,80,104,105]. PCL degrades to form the products that are metabolized via the tricarboxylic acid cycle or are finally eliminated [106]. PCL is a hydrophobic, semicrystalline polymer [104]. Its low melting point (55–60 °C [60]), good solubility, and excellent compatibility with other materials give encouraging prospects for its use in subcutaneous implants [104].

PCL has a relatively long degradation time, ranging from several months to years. The degradation time depends on the polymer molecular weight [104] and the degradation conditions such as temperature, pH, and the presence of enzymes. The degradation time increases as the molecular weight increases. As the molecular weight increases, the chain length and, therefore, the number of ester bonds that need to be cleaved to create water-soluble monomers and oligomers also increases [104]. Slow degradation also results from the hydrophobic character of PCL, which does not allow its structure to be penetrated by water molecules [105]. The rate of ester bond hydrolysis depends on the factors increasing water penetration into the polymer [102].

The slow PCL degradation time distinguishes it from the other polymers such as PGA, PLA, and PLGA [106]. However, PCL is a relatively cheap polymer, which may make it more cost-effective. The long degradation time of PCL allowed examining its medical potential [58] thoroughly.

##### Poly(caprolactone-co-poly(ethylene glycol))

PCL is compatible and forms miscible blends [33] with other polymers. It provides the opportunity to create polymer blends with unique properties and degradation kinetics. It has been found that copolymerization with hydrophilic monomers can significantly increase the rate of PCL degradation [106,107]. For instance, the use of hydrophilic, nonimmunogenic, and nontoxic poly(ethylene glycol) (PEG) allows copolymerization with PCL to create a material with better hydrophilicity and biodegradability [108].

It has been demonstrated that PCL-PEG copolymers have increased biocompatibility compared to the PCL homopolymer [108]. The addition of PEG to PCL shortens the polymer degradation time as a result of increased water penetration and an enhanced hydrolysis rate.

The glass transition temperature (T_g_) and crystallinity of the polymer are the factors that determine the ability of water to penetrate the polymer [102]. High T_g_ correlates with limited molecular motion, low free volume in the polymer, and, consequently, reduced availability for water penetration. Thus, the reduction of T_g_ and crystallinity accelerates the hydrolytic degradation of the polymer. The addition of PEG to PCL-PEG blend results in a lower T_g_ and crystallinity copolymer and shortens the degradation time.

The rate of degradation can also be altered by copolymerization with other lactones, glycolides, or lactides [104]. PCL, therefore, is a highly diverse material and has the potential to be a suitable polymer in the development of implantable drug delivery systems.

##### Polyurethanes (PUs)

PUs are formed by the reaction of diisocyanates with polyols (or equivalent) in the presence of a catalyst. PUs have a wide variety of industrial uses. Much recent attention has focused on their biomedical applications, owing to their biocompatibility, biodegradability, and tailorable chemical and physical forms. Examples of such application areas include antibacterial surfaces and catheters, drug delivery vehicles, stents, surgical dressings/pressure-sensitive adhesives, tissue engineering scaffolds, and electrospinning. This review analyzed selected articles, mainly from the years 2014–2017, in which a diverse range of biomedical applications of polyurethane materials and coatings in cardiovascular products were described [55].

##### Other Biodegradable Polymers

In the field of medical materials technology, there are many less commonly used biodegradable polymers for the delivery of medicines, including poly(amides), poly(anhydrides), poly(phosphazines), and poly(dioxanone) [59,109]. Poly(anhydrides) have low hydrolytic stability, resulting in rapid degradation rates, making them suitable for use in short-term controlled delivery systems [110]. Poly(phosphazenes) have a degradation rate that can be finely tuned by appropriate substitution with specific chemical groups, and the use of these polymers has been investigated for skeletal tissue regeneration and drug delivery [110]. Finally, is poly(dioxanone), which, like PCL, is a polylactin used for purposes such as drug delivery and tissue engineering [111].

#### 3.2.3. Biodegradable Metal Scaffolds

In addition to biodegradable polymers, biodegradable metals are considered to be useful for the construction of biovascular scaffolds. The metal used for the development of biodegradable scaffolds must be not only biodegradable but, also, biocompatible and biocorrosive. The metals meeting these criteria include magnesium (Mg) [4]. Magnesium and its alloys have become the subject of many studies on metallic biomaterials used in cardiovascular stents. Studies over the possibility of using Mg and Mg alloys as implant materials were started in 1878 by Witte et al. [112].

The reaction between Mg and water molecules results in the degradation of magnesium into Mg^2+^ ions and H_2_ molecules, shown as Mg(s) + 2H_2_O (l) → Mg^2+^ + 2OH^−^ + H_2(g)_) [113]:

Mg_(s)_ + 2H_2_O (l) → Mg^2+^ + 2OH^−^ + H_2(g)_ (1)

The degradation of Mg takes between two and 12 months, depending on its composition. Recent studies have reported of 9–12 months of radial support for Mg-based stents [46]. It has been demonstrated that a magnesium stent implanted in an animal model lost its mechanical integrity in 35–36 days, and no evidence of thrombosis was reported [114]. First-generation magnesium-based BRS were noneluting, i.e., they lacked antiproliferative drug release from the stent surface. It was suggested that the emerging electronegative charge during degradation of the metal platform could function as an efficient antithrombotic agent [45].

Recent studies have shown that, in addition to magnesium, iron-based scaffolds also demonstrate the required biodegradability, safety, and efficiency. Tests on animal models showed iron-based scaffold degradation within 28 days of their introduction to the body. Throughout these days, no in-stent thrombosis, excess inflammation, or even fibrin deposition was seen [115]. However, the success of this project needs long-term follow-up studies to analyze the efficacy of the corrodible iron stent [4].

Table 7 shows the research in which the use of magnesium as a construction material for scaffolds is discussed. The table contains examples of other solutions based on biodegradable metals. Some of them have already been launched on the market; others are still poorly researched and require further testing.

The most common allegation against biodegradable metal stents is that they degrade too quickly. There are concerns that the too-dynamic corrosion of the metal core could pose a severe threat to the cardiovascular system [116,117]. Modification coatings have been developed to slow down decomposition. Polymer coatings are usually proposed as an alternative solution to address this challenge [116,117,118,119,120,121]. The study carried out by Jiang et al. showed a positive effect of biodegradable polymeric coating on the reduction of the Mg-based scaffold’s degradation rate. The results encouraged further researchin particular, into PLGA as a potential biomaterial for cardiovascular applications [118].

The improvement of corrosion-resistance Mg and its alloys can be achieved by the already mentioned scaffold surface polymer coating but, also, by appropriate modification of the alloy composition and its microstructure. It has been shown that, in most cases, the applied coatings have an anticorrosion effect on Mg and its alloys [122]. Micro-arc oxidation (MAO) is a surface treatment method for improving the corrosion resistance of Mg alloys [123]

## 4. Drug Release Kinetics

Releasing the correct therapeutic drug dose from a bioresorbable DES is essential for inhibiting smooth muscle cell growth, neointimal hyperplasia, and in-stent restenosis (ISR). In the study [124], the in-vitro release profiles of sirolimus-in-poly (D, L-lactide) (PDLLA) coatings were investigated under various conditions. Firstly, single-layer, bilayer, and various ratios of sirolimus/PDLLA coatings on biodegradable poly (L-lactide) (PLLA) stents and tubes were prepared. There was no apparent delamination or cracking on the stent-coating surface that underwent crimping and expansion. Secondly, the degradation performances of drug-free PDLLA films were investigated to analyze the effects of the changes in molecular weight and mass loss. Finally, the in-vitro sirolimus release profiles of various coating formulas in phosphate-buffered saline (PBS) were studied by high-performance liquid chromatography (HPLC). The results indicated that the profiles exhibited similar two-phase release kinetics, but the initial release rates were quite different. Moreover, coatings with polyethylene glycol (PEG) additives were prepared to assess their controlled release behaviors. The reported research represents a step towards establishing an in-vitro release model, which will be verified in future works after comparison with in-vivo release profiles.

Recently, the biodegradable polymeric matrix used as the kingpin of the local drug delivery system is the center of attention. The work [125] focused on the formulation of the mathematical model elucidating degradation of a drug-loaded polymeric matrix followed by drug release to the adjacent biological tissues. Polymeric degradation is related to mass-preservation equations. The drug release phenomenon is modeled by considering the solubilization dynamics of drug particles and diffusion of the solubilized drug through the polymeric matrix, along with the reversible dissociation/recrystallization process. In the tissue phase, reversible dissociation/association, along with the internalization processes of medicine, are taken into account. For this, a two-phase spatiotemporal model was postulated, which ensued a system of partial differential equations. They are solved analytically with an appropriate choice of initial, interface, and boundary conditions. To reflect the potency of the advocated model, the simulated results are analogized with the corresponding experimental data and found laudable agreement to validate the applicability of the model considered. This model seems to foster the delicacy of the mantle enacted by important drug kinetic parameters such as diffusion coefficients, mass transfer coefficients, particle binding, and internalization parameters, which are illustrated through a local sensitivity analysis.

The aim of the research of Zhang et al. [126] was to investigate the drug-release profiles of biodegradable polymer sirolimus- or paclitaxel-eluting stents with an asymmetrical coating (BPSES-A or BPPES-A) both in vitro and in vivo. As for in vitro, the drug-release profile was characterized by measuring the drug concentration by HPLC over a time-course. In the case of in vivo, a porcine aorta stenting model was employed. The results showed that the drug release rates of BPSES-A and BPPES-A were slower, more stable, and less burst-releasing than those of conventionally coated stents (BPSES-C and BPPES-C, respectively), both in vitro and in vivo. Based on the in-vivo results, the authors concluded that the sirolimus and paclitaxel contents of the local coronary wall were maintained at higher and more effective levels with BPSES-A and BPPES-A compared with BPSES-C and BPPES-C, respectively. The drug levels in the peripheral tissue samples were below the detection levels. These data demonstrated the effectiveness of both sirolimus and paclitaxel as stent-coating agents. They revealed the favorable drug release kinetics and pharmacokinetics of asymmetricalcoated stents compared with conventional coated stents [126].

In the review [126] article by Zhang et al., the currently employed or explored delivery concepts for local intravascular drug delivery with drug-eluting stents (DES) were discussed with a particular emphasis on clinical evidence regarding the desired release profiles. Traditional ideas to control drug releases from DES include diffusion through polymers, polymer degradation, and erosion, as well as dissolution of the particulate drug. Published clinical studies do not always reveal fine mechanistic details. The long duration of release favored for DES and the short period of release sanctioned for drug-eluting balloons require further investigation in experimental studies and clinical trials.

## 5. DES Coating Techniques

Implantation of drug-releasing stents (DES) by percutaneous coronary intervention is the most common treatment option to restore blood flow to the obstructed vascular system. Many devices currently used in a clinic or under examination in research laboratories are manufactured with a variety of coating techniques to create incorporated drug release platforms. These coating techniques offer various benefits, including ease of use, the expense of equipment, and design variability [34]. Several types of stent-coating techniques are proposed, each posing its benefits and drawbacks that must be considered when choosing between them. It may include dip coating, electrotreated coating, plasma-treated coating, and spray coating.

Since the stent surface plays a critical role in the success of the implantation [127] it should be biocompatible and show anticoagulation and antithrombotic, anti-inflammatory, and proendothelializing effects after implantation. Thus, surface modifications must meet the following requirements:

- inhibition of an inflammatory reaction for impeding the thrombosis formation,
- inhibition of excessive SMCs proliferation and preventing intimial hyperplasia,
- fast endothelialization from the early time of implantation to promote the creation of an endothelial layer on the stent surface within one month; a quick endothelialization process is essential to decrease the risk of thrombosis to the least amount, and
- avoiding adverse material-tissue interface interactions; it is necessary for the surface to be biocompatible, especially after complete drug elution [127].

In the literature, there are two succeeding coating techniques for surface modification: physical and chemical.

The burst-released type proposed by Hu et al., 2015 [10] is an example of the drug-coating technique where the medicine molecules are firmly bound via chemical bonds to the surface, empowering the DES system to release a drug in a sustained manner. However, generating groups that chemically bond to the surface usually requires unique treatments (anodic oxidation, acid/alkaline treatment, or silanization) [128].

In the physical coating method, a liquid solution is applied by dipping, spraying, or brushing. Additionally, the layer-by-layer (LBL) assembly technology is used, in which a nano-thin layer of polyanions and polycations can be formed on the charged surface from an aqueous bath [129].

The frequently performed stent-coating techniques such as dip coating, electrotreated coating, plasma-treated coating, and spray coating are presented and briefly discussed in the next sections.

### 5.1. Dip Coating

**Figure 3:**
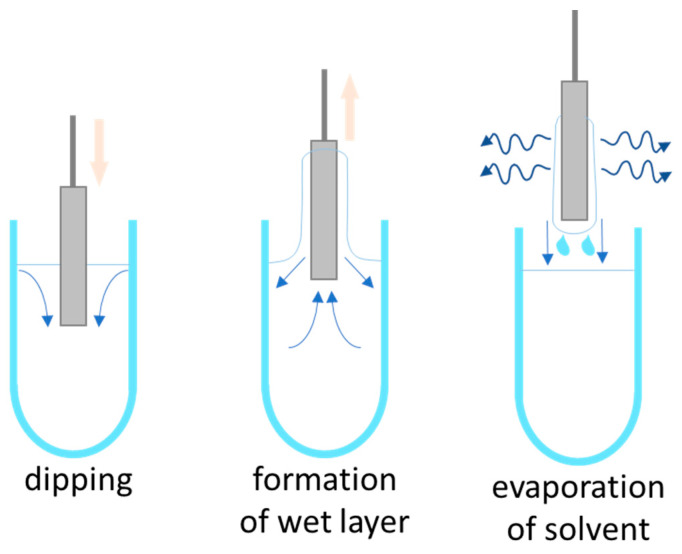
Dip-coating schematic [130].

Dip coating is a basic technique that does not require extensive machinery or time. It involves submerging the stent in a solution of typical drugs and/or polymers in a solvent. The stent is then left to dry, allowing for evaporation, in the air or an oven, as shown in Figure 3 [130]. While using this method, the polymer, drug, and concentrations can vary. For instance, Jang et al. coated stents with either a low dose or high dose of curcumin without the presence of any polymer.

### 5.2. Electrotreated Coating

To expand the available options for coating techniques, researchers have begun incorporating electrical stimulus into stent-coating techniques to assist in drug/polymer deposition onto the stent surface or to increase polymerization on an already deposited drug-release layer.

**Figure 4:**
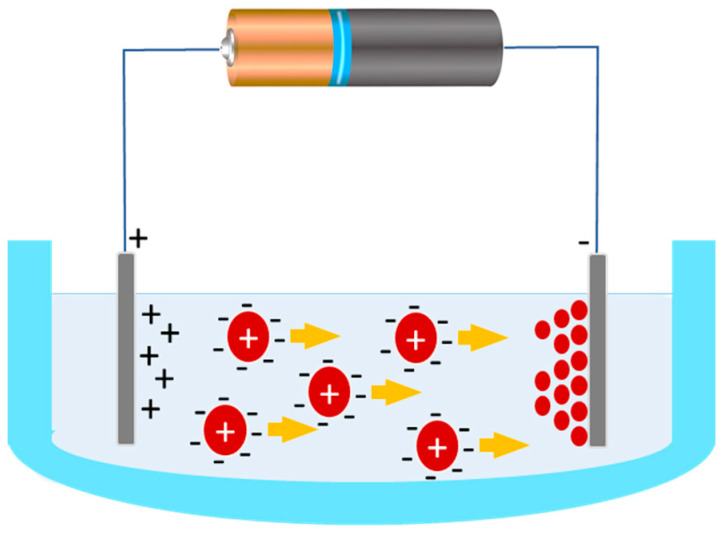
Electrophoretic deposition (EPD) in a solution schematic [132].

Electrophoretic deposition (EPD) is a technique that uses an electric field, either in a dry environment or soluted, to attract charged particles onto the stent surface, resulting in the formation of a drug-release layer. A scheme of the EPD apparatus is shown in Figure 4 [131].

**Figure 5:**
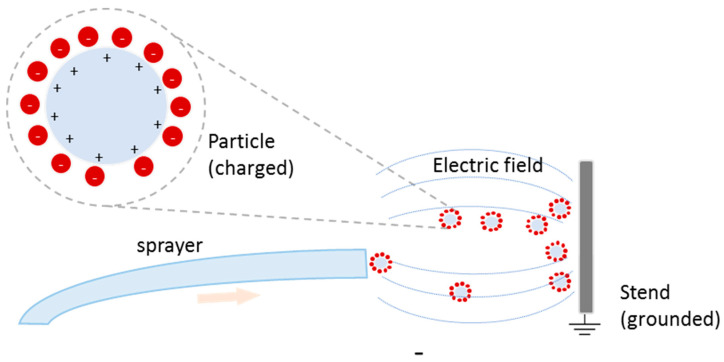
Electrostatic dry powder deposition schematic [133].

Utilizing electrostatic dry powder deposition (Figure 5), Nukala et al. coated stents with sirolimus-loaded PEVA and PBMA microparticles, comparing the resulting release profiles with the Cypher stent release profile (Cordis Corp., Hialeah, FL) [133]. Though this design used the same polymers as the Cypher stent, it exhibited a three-day burst release of 50% compared to 35% release by Cypher, as well as a 100% total release after 25 days compared to 85% release by Cypher [133].

Designing another DES coated using multiple techniques, Liu et al. deposited *N*-nitrosomelatonin (NOMela)-loaded PLGA nanoparticles using EPD onto SS 316L stents, then used dip coating to create a diffusion barrier of collagen [134]. This work studied the release profiles of a model hydrophobic and hydrophilic drug after immersing the stent in PBS (pH 7.4) with and without 5% (*v*/*v*) Tween 80, respectively. The samples, including a top collagen layer, showed a burst release of 50–70% in 24 h, with another 20% of the encapsulated drug released between day two and day 14 [134]. Overall, the integration of an electrical stimulus to aid in stent coating presents itself as an exciting development. Still, the safety and efficacy of the electrotreated stents have not been evaluated in clinical models, only in noninferiority animal models.

### 5.3. Plasma-Treated Coating

Most recently, researchers have started to include plasma treatments into their coating designs to strengthen the chemical bonds in the drug-release layer via polymer crosslinking. This technique involves exposing the base metal or polymer-coated stent surface to a gaseous plasma beam for varying lengths of time. A group led by Hagiwara evaluated the potential of this as a release platform for DES by plasma-treating silicon wafers coated with curcumin-loaded PEVA, studying specifically the effects of exposure to argon, oxygen, and nitrogen plasma over time. They found that untreated stents released up to 120 μg of the drug in 14 days, while highly treated samples only released between 5 and 50 μg (depending on the gas) in the same time frame [135]. While this technique presents a relatively simple way to increase the intermolecular strength of the stent coating, it is still in the fledgeling stages of development.

### 5.4. Spray Coating

**Figure 6:**
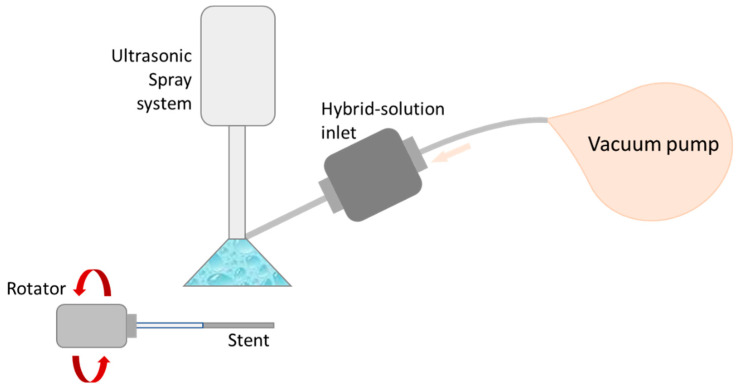
Spray-coating system schematic [136].

The most commonly used stent coating techniques include ultrasonic atomization, electrodynamic jetting, and air brush coating. These techniques use devices that spray polymer and drug solutions (using various solvents) onto a stent, enabling a consistent deposit of uniform drug-release layer(s) onto the stent surface. For this discussion, a set of these techniques will be referred to as spray coating. Spray coating can be performed using several systems, one of which is described in Figure 6. This technique allows for a higher variability of coating designs, resulting in better optimization of the release profile. In general, this technique exhibits a logarithmic release curve characterized by a burst release, caused by the presence of the drug at the boundary layer between the stent and the surrounding vascular environment, followed by a slower release of the drug to enable long-term therapeutic effects.

Spray-coating techniques manufacturing DES and studying drug-release profiles are important directions in the area of cardiac research. This is the most straightforward technique for scaling up a high volume of consistently coated stents. The difficulty with evaluating this technology is that the immense number of variables manipulated makes broad comparisons between the individual designs nearly impossible.

### 5.5. Drug Delivery Mechanism and Effective Parameters

It is imperative to understand the mechanism of drug delivery to use the right choice of drug for a time-ordered release [136]. Polymeric systems have been known as efficient drug carriers for two reasons, including providing a framework for controlled drug release and protecting the drug from degradation before it acts effectively [137]. The mechanism of drug release from the polymer substrate can be classified based on the drug polymer binding into two significant mechanisms: physical and chemical. Physical mechanism refers to the drug release through a permanent polymer layer, dissolution or degradation of the polymer, the permeation pressure, and through an ion exchange process. The chemical mechanism is due to the breakage of covalent bonds, which happens as a result of chemical or enzymatic degradation [10].

The initial drug-polymer system was based on nonbiodegradable polymers where the drug diffusion process was controlled by the concentration gradient. Later, biodegradable polymers were used as the significant drug-eluting system [132]. There are three major mechanisms based on the type of polymer in which the drug is released, including diffusion (for permanent polymers), swelling (for polymers with the swelling ability), and erosion (for biodegradable systems) [132]. The primary controlled-release devices are classified into reservoirs and matrix systems. In reservoir systems, the drug is located in the center and is surrounded by a polymeric membrane through which it diffuses out. In addition to the membrane form, reservoirs can also have the form of microcapsules or hollow fibers [138]. Another way of a polymeric system to carry the drug is a matrix device throughout which drug agents are distributed. Matrix devices are more favorable to use as drug carrier systems, for they prevent any burst release and are easy to manufacture compared to the reservoirs. In the diffusion-controlled order, it is vital for the system to be stable when placing it in the biological environment, i.e., not changing its size either through swelling or degradation.

More importantly, the polymer-drug combination should not induce any change in the polymer structure. At the same time, the drug must be able to diffuse through the polymer pores or macromolecular structure at a sufficient rate [139].

**Figure 7:**
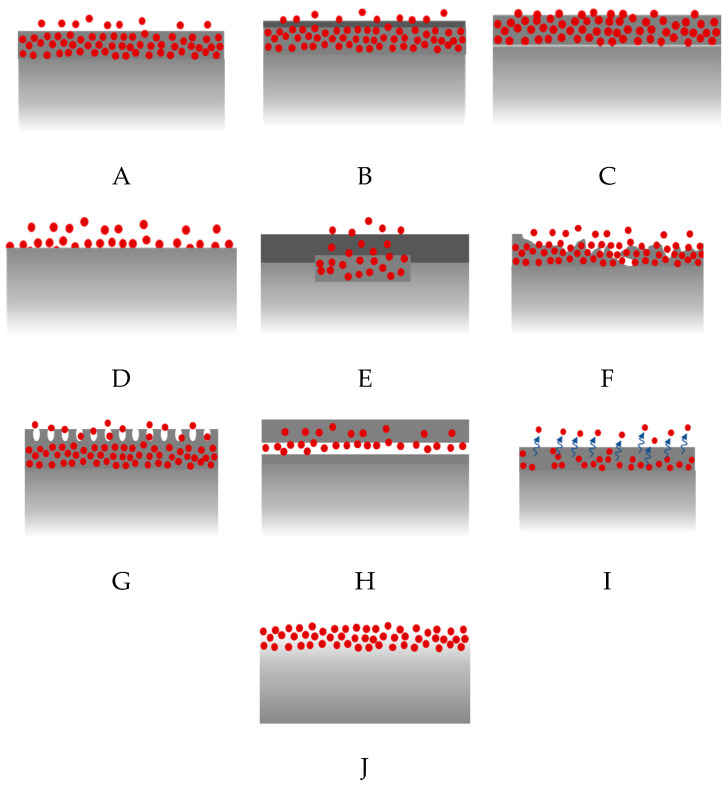
Different types of stent-based drug delivery systems: (**A**) drug released by diffusion from the polymer, (**B**) drug released by diffusion through a rate-limiting coating, (**C**) drug released by swelling of the layer, (**D**) drug release directly from the surface, (**E**) drug loaded in pore or reservoir in-stent, (**F**) drug release by the erosion of the polymer coating, (**G**) drug loaded in a nanoporous reservoir in a sheet, (**H**) drug packed between coating layers, (**I**) drug released by hydrolysis or enzymatic action from a polymer, and (**J**) bioerodable polymer-coating stent.

The schemes of drug release via the surface in five marketed stents, including two permanent polymer-coated, one biodegradable polymer-coated, and two polymer-free stents, are illustrated in Figure 7.

In swelling-controlled systems the drug-release device is initially dry, but when placed in the body, it absorbs water and swells. The advantage of this system is that the drug release starts immediately after the device is placed in an appropriate biological environment [132,140].

In the swelling process, the polymer-free volume increases, and the drug diffuses through the swollen network into the site of injury [137]. In contrast to the permanent polymeric drug-carrier systems that do not change their chemical structures during drug diffusion, biodegradable polymers degrade within the biological condition after a particular time. By degradation, these polymeric drug-eluting systems eliminate the need to be removed from the body after releasing active pharmaceutical agents [79]. For this superior property over nondegradable polymers, a great deal of research has been conducted on degradable- and erosion-controlled systems [137]. There is a difference between degradation, which is a chemical process, and erosion, which is a physical phenomenon. Decay can be classified into surface erosion and bulk erosion; the chemical structure of the polymer dominantly determines the erosion phenomenon. When the rate of erosion exceeds the rate of water absorption by the bulk of the polymer, surface erosion occurs. On the other hand, bulk erosion is the drug-controlled mechanism where the rate of water permeation into the bulk is higher than the rate of decay [141]. Most biodegradable polymers for delivery systems undertake bulk erosion (polylactide and polyglycolide polymer families) [137].

The main advantage of the physical mechanism is that it can be controlled with the designed stenting system. In other words, the stenting system has predetermined kinetics that can be adjusted to a preferred one by changing the efficiency parameters. In the chemical drug delivery mechanism, grafting drug molecules could result in new chemical bonds that are the disadvantageous to the system. The chemical mechanism itself is based on the breaking of chemical bonds that bind drug molecules to the system and creates new chemical bonds, making the breakage much trickier. In some studies, it is far preferred to work with a simple physical mechanism for controlled drug delivery [42]. Many agents with anti-inflammatory or antiproliferative properties have been incorporated on the stent surface and tested clinically (Table 8 and Table 9). Many of the agents listed in the tables have more than one mechanism of action. The general mechanism of action for most of these drugs is to stop cell cycle progression by inhibiting DNA synthesis.

## 6. New Stent Systems

### 6.1. Shape-Memory Stents

Another type of stent that might play a part in future trend as the primary technique is the shape-memory stent. It has the ability of self-expansion in the range of body temperature in an ideal situation [142]. Shape-memory polymers (SMP) are materials that react to stimuli that change their shape in response to an external factor. They contain a two-phase shape transition. In the first phase, the polymer is fixed in a brief form. In the second, the polymer is boosted by an external factor to recover its permanent shape.

### 6.2. Polymer-Free DES

Despite the favorable effects of DES, clinical studies have proven the inflammatory triggering effects of toxic ions generated from the degradation of polymeric coatings or degradable metal or metal alloys as a surface coating [143]. An option to ultimately get rid of polymers as the drug-carrier is to develop a polymer-free stent. This alternative should be able to preserve the functionality of polymeric DESs, including carrying drug molecules, binding the drug to the stent, and controlling the drug release rate at a suitable rate [144]. More importantly, carrier-free stents need to be biocompatible to adapt to the tissue surrounding. In comparison to the polymeric coating as the drug-loading platform, polymer-free stents are expected to have a faster drug elusion rate, which might have an adverse therapeutic effect. However, the efficacy and safety of the latter ones seem to be comparable to the first-generation DES.

Although polymer-free stents have been performing well in preclinical and clinical trials, these stents do not outperform second-generation DES yet. There are seven coating technologies for polymer-free stents, including direct coating, crystallization of the drug, nano- and microporous surfaces, inorganic porous coating, macroporous drug reservoirs, the coating of nanoparticles, and self-assembled monolayers [144].

**Figure 8:**
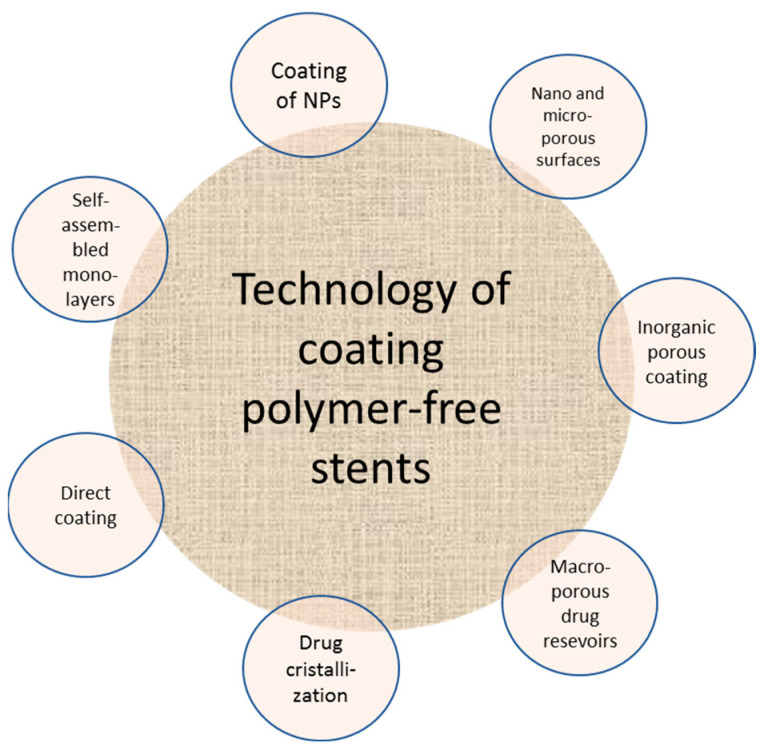
Schematic representation of the techniques used to manufacture polymer-free DES [42]. NP: nanoparticles.

Thiruppathi and Mani [145] named the same list of seven coating technologies in a different manner, including molecular coatings [146], self-assembled monolayers [147], micro-rough surfaces [148], porous surfaces [149], textured surfaces [150], and reservoirs [151]. A schematic representation of these coating techniques is shown in Figure 8.

Thiruppathi and Mani [145] conducted comparative research on stents with a polymer-based-PLGA platform and a polymer-free platform (a polymer-free phosphoric acid platform). A CoCr alloy surface was used as the stent and an auxiliary platform for loading vitamin C (l-ascorbic acid, l-AA). 1-AA is an antiproliferative drug that promotes endothelial cell growth faster than antiproliferative conventional medicines. The potential ability of vitamin C (l-AA) to act as a therapeutic drug over antiproliferative drugs such as sirolimus for encouraging endothelial cell growth has been demonstrated in their previous study [152].

Despite the successful introduction of l-AA coating onto CoCr alloy surfaces, the results of drug releases from two distinct platforms of polymer-free and polymer-based platforms have shown significant differences. l-AA was burst-released from the polymer-free CoCr alloy surface for one h. Whereas, l-AA was sustained-released from the PLGA-coated CoCr alloy surface for 24 h [145].

The application of nanotechnology, through the use of mesoporous silica nanoparticles (MSN), appears to be an excellent solution for the creation of polymer-free drug-eluting stents. Stent modified this way have undeniable advantages, such as a tunable pore size, a high specific surface area, large pore volume, and biocompatibility to the tissue [153,154,155], which makes them a handy tool in vascular surgery.

In a study by Wang et al. [156], a novel polymer-free DES stent was constructed, making use of magnetic mesoporous silica nanoparticle MMSNs and carbon nanotubes. The nanostructured coating on the stent platform resulted in a new polymer-free stent that exhibited excellent mechanical flexibility and blood compatibility, with a satisfactory drug-releasing profile.

A promising report was produced by a research [157,158] in which a nano-thin microporous hydroxyapatite surface-coated stainless steel stent was used as a polymer-free DES for releasing sirolimus. The low-dose drug-loaded polymer-free stent showed satisfying results for one-year clinical trials. Recently, a report has been published on the drug delivery and capability of DES-based crystallized coating [15] using the polysaccharide top coating applied onto the rapamycin (RM) crystalline layer. The top coat was a protective layer for the crystalline poly to suppress delamination during the stent crimping and expansion. The crystalline coating enhanced the quality of the carrier-free stenting system regarding the physical, mechanical, and chemical stability of the stent [15].

The presented efforts contributed a lot to the development of polymer-free stents. However, there is still a need to design new materials and coating structures necessary to improve the safety and functionality of stents.

Nanotechnology could be beneficial in this field. The literature review shows that this type of modification of stents has brought many advantageous features. The duration of drug release from the stent has been improved, although the mechanical integrity required for the stent backbone has been problematic in most cases [159]. However, it is worth noting that their long-term safety distinguishes polymer-free stents compared to traditional DES-coated stents [15]. There is still no guaranteed success, though, and more research studies need to be developed.

### 6.3. The Future of DES

In recent years, many research groups have been working to improve the DES available on the market. The design of the third-generation DES focuses mainly on solving problems related to the use of older DES, primarily focusing on the prevention of restenosis, acute thrombosis, and LST (Leucocyte Specific Transcript) [160]. The demand for the third generation of stents is still enormous, because stent implantation is a widely used, rapidly developing technique in the clinical treatment of coronary artery disease.

#### 6.3.1. Solutions for Cardiac Patients

Although the patient’s cardiovascular state depends on their age, gender, and health condition, the development of customized individual stents has been limited. Thus, a patient-specific stent manufacturing system should be designed for more successful stent therapy. In the [161] study, a 3D-printed PLA biodegradable polymeric stent was prepared using polydopamine (PDA), polyethyleneimine (PEI), and heparin (Hep) chemistry to prevent restenosis and thrombosis with anticoagulation and excellent blood compatibility. The physicochemical characterization indicated that pristine PLA substrates were well-modified, as the abundant amine surface allowed for coating with a large amount of heparin. From in-vitro and ex-vivo analyses, heparinized 3D PLA stents showed excellent thromboresistance and hemocompatibility, modulation of the smooth muscle cell (SMC), and endothelial cell (EC) proliferation. In an in-vivo study, the heparinized 3D PLA stent showed the most extensive lumen area with the least neointimal hyperplasia and without atherosclerosis or thrombosis. All these assessments confirmed that the presented innovative strategy proposes a useful model as a method of preparing a fully biodegradable individual stent for successful implantation therapy. It would be widely used in clinical cardiac applications [160].

#### 6.3.2. Solutions for Cardiac Patients with Diabetes Mellitus

The high lifetime risk of vascular disease is one of the critical issues that plague patients with diabetes mellitus. Systemic oral vildagliptin administration favors endothelial recovery and inhibits smooth muscle cell (SMC) proliferation. However, the localized release of vildagliptin in diabetic vessel damage has seldom been investigated. In the study [161], sufficient vildagliptin concentrations were delivered for more than 28 days from the nanofibrous membrane coatings on the surfaces of the stents in vitro and in vivo. It was shown that the vildagliptin-eluting PLGA membranes greatly accelerated the recovery of diabetic endothelial and reduced SMC hyperplasia. The type I collagen content of the diabetic vascular intimal area that was treated by vildagliptin-eluting stents was lower than that of the non-vildagliptin-eluting group. In this work, nanofiber-eluting stents loaded with vildagliptin, a dipeptidyl peptidase-4 enzyme (DPP-4) inhibitor, were fabricated to treat diabetic vascular disease. The poly (D, L)-lactide-co-glycolide (PLGA) and vildagliptin were mixed using a hexafluoroisopropanol and electrospinning process to prepare the nanofibers. In-vitro and in-vivo release rates of the vildagliptin were characterized using high-performance liquid chromatography [161]. The experimental results revealed that stenting with vildagliptin-eluting PLGA membranes could potentially promote healing for diabetic arterial diseases.

#### 6.3.3. Stents in the Management of Chronic Rhinosinusitis

Drug-releasing stents are also used to treat other conditions. Campbell et al. in the review [162] discussed the literature assessing the use of drug-eluting stents in the management of chronic rhinosinusitis. In the surgical management of chronic rhinosinusitis, medical therapy is often used to prevent postoperative complications such as nasal mucosa adhesions. These complications often require painful debridements in the clinic or revision surgery. The application of systemic steroids is not free of risk, and topical steroids are not ideal, as the duration of mucosal contact and exact dosage are unknown. The penetration into gravity-dependent sinuses is suboptimal [162].

Chronic inflammation and infection of the nasal sinuses, also referred to as chronic rhinosinusitis, severely affects patients’ quality of life. Adhesions, ostial stenosis, infection, and inflammation relapses complicate chronic sinusitis treatment strategies. Drug-eluting stents, packings, or implants have been suggested as reasonable alternatives for addressing these concerns. Parikh et al. in [163] reviewed potential drug candidates for nasal implants, as well as formulation, optimization, and characterization methods. The advantages, limitations, and future considerations for additional research in the field of nasal drug implant development were discussed [163].

#### 6.3.4. Stents in Urological Procedures

Wiesinger et al. [30] in a review summarized the most recent evidence on the use of ureteral stents, including the use of different materials and the treatment of stent-related symptoms. According to the authors, after more than five decades of using stents, there are promising advancements in their designs and materials, aiming to maintain their patency and control stent-related symptoms. Long-term metallic stents and coated stents are good options that should be considered in selected patients. Biodegradable stents are promising but not mature enough yet. Pain medication, alpha-blockers, and antimuscarinic medications are still frequently used and necessary. Treatment combinations can result in better outcomes than monotherapy [30].

#### 6.3.5. Herbal Stent Coatings

Lukman et al. in [31] presented the results of a study using herbs as alternative coating materials for DES to overcome the long-term complications of delayed endotheliation, delayed wound healing, and late-stent thrombosis. As reported in the literature, potential herbs that can be used for DES coatings are as follows:

- Tripterygium wilfordii [164],
- Atractylodes macrocephala Koidz [165],
- Gastrodia elata Blume [165],
- Citrus unshiu Marcow [165],
- Poria cocos Wolf [165],
- Crataegus pinnatifida Bunge var. typical C. K. Schneider [47],
- Siegesbeckia pubescens Makino [165],
- Coptidis japonica Makino [165], and
- Magnolia Cortex [166].

## 7. Drug-Eluting Balloons (DEB)

A drug-eluting balloon (DEB) is a non-stent technology in which the vessel wall recieves the effective homogenous delivery of antiproliferative drugs from an inflated balloon. It is done to restore the luminal vascularity to treat atherosclerosis, in-stent restenosis, and reduce the risk of late-thrombosis without implanting a permanent object. The balloon technology relies on the concept of targeted drug delivery, which helps in rapid healing of the vessel wall and prevents the proliferation of smooth muscle cells [14].

DEBs may stand for an alternative to DESs in some clinical situations. The rationale for the development of DEBs derives mainly from the limitations of DESs [14]. The initial enthusiasm for reducing the incidence of restenoses after DES was weakened by reports indicating the risk of late-thrombosis after antimitotic drug-releasing stents. Although certainly, for many years, DES will remain an essential tool in the treatment of coronary artery disease, it should not be forgotten that their use is not entirely effective and safe. Apart from the risk mentioned above of thrombosis, the phenomenon of restenosis cannot be ignored, although it occurs on a smaller scale than when using BMS [167,168,169,170,171].

DEBs ensure even distribution of the drug for a short period of balloon inflation and contact with the vessel wall and do not increase the risk of chronic inflammatory reaction. Despite the differences in the construction of individual balloons, they share common features. In DEB systems, mainly lipophilic drugs are used for better tissue absorption, which shortens the time to fill the balloon and extends the effect after emptying, preventing the premature flushing out of the active substance [172].

Like DES, DEB systems are the subject of numerous scientific and clinical studies, as evidenced by systematic publications. Table 10 summarizes the review papers that have emerged in the last five years on this subject.

Presented quotations discuss various aspects of DEBs, including the materials used to produce balloons, coatings, active substances, coating techniques, and the efficacy of finished products evaluated based on clinical studies [14].

In general, research work in the field of DEB focuses on the continuous improvement of their performance and safety by improving the device components, such as:

- materials used to make balloons,
- types of medications used for coating, and
- coating techniques.

### 7.1. Balloon Materials

To meet the high expectations of physicians, balloon catheters should have adequate flexibility, high mechanical strength, and be thinly walled. The balloon stent should also have the capacity to fold three–seven times to inflate and pressurize the plaque [193]. In response to these requirements, catheters of various lengths and diameters, as well as different designs, are available on the market. These include offset, multilayer, spiral, and multi-channel balloons.

The first angioplasty balloon used by Gruentzig was made of flexible polyvinyl chloride. Currently, balloons are made mainly of thermoplastic polymers (Table 11) [194]. There are also balloons constructed of crosslinked polyethylene, polypropylene, polyamides, polyimides, polyesters like polyethylene terephthalate (PET), polyethylene (naphthalene dicarboxylate), and nylon [195]. There is still a constant challenge to develop a balloon with the ability to control its pushiness, trackability, and crossability [14].

### 7.2. Coating Materials for DEB

Polymeric materials are used to coat the balloon to hold the drug for controlled release. Polymers adhere to the balloon before inflation and attach to the vessel after inflation to decrease thrombosis [56]. Any material that is biocompatible, biodegradable, and bioabsorbable with an adhesive nature can be used as a matrix to coat the DEB [196]. The effectiveness of DEB depends, among others, on the type of drug used for coating and the balloon-coating technique. Bioadhesive polymers help by attaching to the endothelium and mucus layer to form entanglements through hydrogen bonding, enabling the drug to be held for a certain period and be released in a controlled manner [197]. Various polymers have been used to date: (1) biodegradable polymers, such as methacrylate, acrylate, polyethylene glycol, or acrylate vinyl pyrrolidone; (2) biocompatible polymers, such as carbopol polymers, carbopol poloxamer gels, hydroxypropylmethylcellulose, guar gum, sodium carboxymethyl cellulose, polyvinyl pyrrolidone, hydroxypropyl cellulose, polycarbophil, polyacrylic acid, chitosan, starch, alginate, and their copolymers; (3) bioadhesive polymers, such as polylactic-co-glycolic acid (PLGA); and proteins and carbohydrates, such as gellan and gelatin, xanthan, mannitol, and chitosan [198,199].

### 7.3. Therapeutic Substances

Several therapeutic substances can be applied to the balloon catheter surfaces. The agents can be antiplatelet, anti-inflammatory, antihyperlipidemic, antiproliferative, and antithrombogenic. Examples of each type of drug and their mechanisms of action are described in Table 12. Among all agents, paclitaxel and sirolimus are the preferred drugs to be applied to the balloons [14].

The drug release must be maintained in place to achieve a therapeutic effect. Moreover, no local or systemic toxicity may occur, and the delivery mechanism should not stimulate restenosis [14,200].

Controlling the release of drugs into the wall of a weathered vessel, when infalting the balloon, is a vital issue in the DEB design process. The critical parameter of the safety and efficacy of the drug is that it should be released at a desirable rate and without a loss of effectiveness. However, it has to be noted that DEBs are unable to secure a long-term sustainable controlled release of the active substance.

### 7.4. Excipients

Currently, DEB developers are proposing to use various biocompatible excipients to cover balloons to transfer drugs to arterial vessels more efficiently. Understanding the complex interaction between the shell and the artery, between the shell and the balloon, and the variety of administration sites has led to the development of highly specialised DEB [201].

The research of Chang et al. [201] was focused on two clinically significant DEB excipients: urea and shellac. The authors used uniaxial mechanical testing, scanning electron microscopy (SEM), and biophysical modeling based on the classic Hertz theory to elucidate how coating a microstructure governs the transmission of forces at the coating-artery interface. Researchers have conducted a series of experiments, including compression tests to assess strength, scanning electron microscopy to study the intrinsic coating structure, and cell cultures to quantify the drug toxicity. They also developed a biophysical model to illustrate the interactions between the balloon coating and the artery during balloon angioplasty. Researchers discovered that microscopic particles of some layers are needle-shaped and some are spherical and that these shapes determine the mechanics of the coating’s contact with the artery, which in turn affects the amount of drug transfer to the arteries [201].

The methods of placing the drug on the balloon surface can be varied. Thus, depending on the model of the balloon and the technology used, there can be found: (1) ballons with the drug applied in an unbound form, (2) balloons covered with a protective layer preventing the premature rinsing out of the drug, and (3) balloons in which the drug is combined with an excipient facilitating its distribution on the surface of the vessel [202]. Excipients are the key to increasing the solubility of the drug, as well as its transfer and absorption [203]. They are also responsible for the mechanism of action of the balloon and have a direct impact on the drug release pattern. The dispersion of drugs in excipients allowed controlling the solubility of hydrophobic drugs in [14].

There is a variety of excipients used today, such as iopromide, urea, dimethyl sulfoxide (DMSO), shellac, polysorbate and sorbitol, butyryl trihexyl citrate (BTHC), and polyethylene glycol (PEG) [204,205,206,207,208,209]. Coatings can be composed of several excipients and can even be applied through layering approaches acting to protect drug loss during tracking, to modulate drug burst, and to provide a controlled drug release [210,211,212].

Iopramide, urea, shellac, and butyryl-trihexyl citrate are some examples of commonly used excipients [213]. Urea is a hydrophilic agent that helps lipophilic moieties or drugs to permeate the arterial wall [214]. Iopramide can be used as a hydrophilic spacer. Other excipients used are lipophilic lubricants like talc, magnesium stearate, and fatty acids to decrease the friction between the balloon and the polymer layer or the vessel [215,216]. Radiospacer materials can also be used to diagnose, locate, and control the movement of the balloon in the vessel [217] (Table 13).

Pharmacokinetics and the functionality of the transport and release of drugs are strongly regulated by the way the additives are placed on the catheter surface. The coating methods include dipping, air spraying, ultrasonic spraying, micro-pipetting, and others [220]. Each of these techniques leads to different coating morphologies and/or drug-elution properties. For example, dipping techniques yield smooth surfaces, but the deposition at balloon folds is highly variable. Ultrasonic air spraying, on the other hand, can cause micro-cracks on the surface, causing the coating to become less stable and result in easier elution of the drugs from the lining to the vessel wall. These coating methods and their effects on the pharmacokinetics, however, are still not well-described in the literature [203].

Auxiliary substances for the transfer and retention of antiproliferative drugs have been used, among others, in DEB systems modified with paclitaxel. It is known that excipient substances play a vital role in the design and functionality of DEB. Still, the methods of coating balloons with excipients and antiproliferative drugs remain poorly explored. The aim of the research conducted by Turner et al. [203] was to develop methods of coating DEB with various excipients and to assess the functionality of such modified DEB [203]. Then, the authors assessed ex vivo the stability of the coating and the transfer of various types of drugs. The application of the methods presented here may lead to the discovery and development of new DEBs with great potential in the process of the delivery and release of drugs.

### 7.5. DEB Coating Techniques

Scientists believe that improving balloon coatings can lead to better designs and improved outcomes. Recently, Chang G.H. et al. [201] examined ballon coatings on a microscopic level, in search of more effective alternatives for arterial diseases.

**Figure 9:**
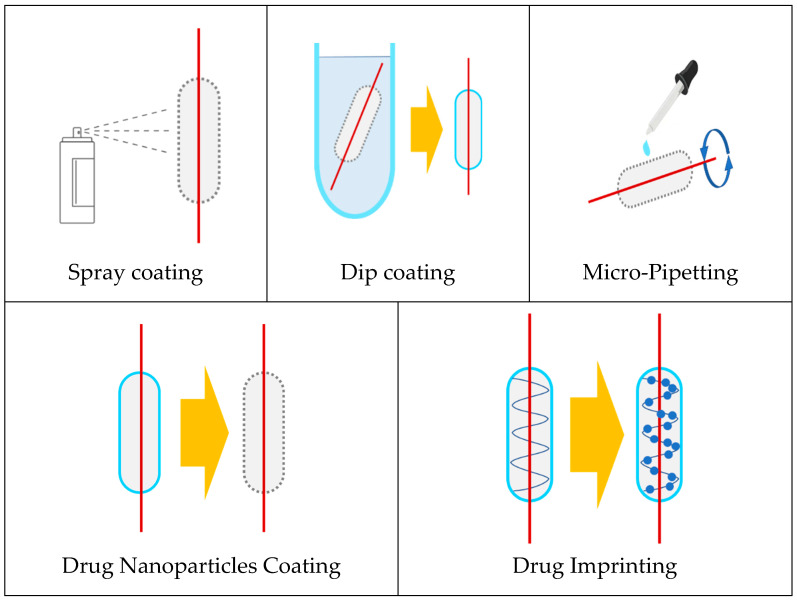
Various coating techniques applied to load drugs onto a balloon [14].

There are different methodologies for loading the drug onto a balloon, like spraying, dipping, micro-pipetting, applying nanoparticles, and imprinting the medicine onto the rough surface of the balloon (Figure 9) [221].

Many innovative coating techniques are known in the literature, many of which have also been patented. Innovative coating methods offer a better-controlled mechanism of drug release increasingly, compared to other models of balloons not long ago used for continuous drug release. The solutions proposed are microcapsule coating, hydrogel coating, polymer-free coating, immediate-release coating, bioadhesive coating, and multiple layer coating.

#### 7.5.1. Microcapsule Coating

The technique proposed here is used to produce a balloon catheter covered with a sticky matrix of microcapsules as a reservoir for the administration of drugs. The surface of the balloon is covered with a layer of microcapsules containing either a single drug or a specific combination of drugs. Drugs adhering to the surface of microcapsules are released into the vessel when the balloon is filled and stretched.

A sheath with a longitudinal weakness is applied over the viscous matrix. When the balloon is stretched inside the vessel, the viscous adhesive cracks, releasing the drug from the microcapsules into the vessel walls [222,223]. There are solutions in which the microcapsule layer is secured with a protective coating to prevent its abrasion. The sheath is removed when the balloon is placed and inflated at its destination.

#### 7.5.2. Hydrogel Coating

Another new solution presented below is the modification of balloon surfaces with hydrogels as a carrier layer for various types of medicines. This type of layer contains a large amount of water and is highly hydrophilic, which improves its bioadhesion. Stankus et al. proposed hydrogel-coated balloon catheters as reservoirs for CAD drugs [224]. The coating consists of a hydrogel polymer and an anti-inflammatory with monoclonal antibodies loaded into the hydrogel matrix. The balloon is coated with three layers, namely the outer, middle, and inner. The outer and inner layers are occlusive layers, which do not contain any therapeutic agent. The layer containing the therapeutic agent is the middle layer [225].

#### 7.5.3. Polymer-Free Coating

It has been reported that bioadhesive polymers remain at the site of the inflated balloon for fifteen days to one month and later degrade [200]. As previously mentioned, biodegradable polymers and their decomposition products can irritate the surrounding tissues while in a vessel. Considering this, a polymer-free coating approach was developed by Speck [226]. This invention concerns balloon catheters coated with “-limus” and/or butylated hydroxytoluene solutions. The device is first coated with the drug and then with the antioxidant in two separate layers, so that the two layers are not homogeneously mixed [218]. Paclitaxel and a shellac solution were applied to the balloon surface using the mentioned technique [227]. Similarly, rapamycin and a shellac solution were also sprayed for the coating [228]. A lipophilic antioxidant was also tried for the surface on the balloon. Catheters coated with the drug and additionally with lipophilic grease were prepared to reduce the friction force. The role of the oil was to store the medication in addition to its sliding function [215]. Balloon catheters partially or entirely covered with paclitaxel and lipophilic excipient are known. The preparation is then coated two to three times on the balloon, forming a multilayer coating.

#### 7.5.4. Immediate Release Coating

Pacetti and Stankus introduced a new type of coating containing a biodurable polymer with a bioactive agent, which upon exposure to the physiological conditions, showed the burst release of the bioactive agent. The maximum elution of the agent was observed within 60s of insertion [199]. Stankus et al. developed a coating technique consisting of a single polymer layer with two tunable solubility profile drugs. In this solution, there were two layers of the balloon, each containing an excipient and another medicine [216]. Both the drugs had different dissolution rates during inflation, which led to the better bioavailability of medicines for therapeutic action.

#### 7.5.5. Bioadhesive Coating

Rowe invented another method for the application of a therapeutic agent with the help of a catheter driving it into the internal tissue of the vessel. The therapeutic agent is mixed with a controlled-release carrier that stays for a more extended period and is biodegradable. This balloon catheter is used along with a stent [229].

Roorda invented a biocompatible carrier to deliver a therapeutic agent through a balloon catheter containing a bioadhesive material [196]. The balloon consists of a single bioadhesive layer carrier that includes the therapeutic agent. The carrier has a higher affinity for the vessel wall than the balloon catheter. The invention describes a single coating layer containing the therapeutic agent and gelatin. The therapeutic agent is embedded in a gelatin matrix. The gelatin starts dissolving once the catheter reaches the desired site and releases the therapeutic agent [219].

#### 7.5.6. Multiple-Layer Coating

Christiansen developed multiple layers of polymer coatings with inner and outer membranes. The inner membrane surrounds the balloon, and the outer balloon membrane surrounds the inner membrane. The outer membrane is more compliant and porous than the inner one, enabling it to release the therapeutic agents into circulation [226]. The two-layered coating contains a therapeutic agent embedded in one layer of the bioadhesive polymer, which is capable of adhering to the blood vessel. The bioadhesive layer is made up of fibers [230]. The second layer formed in between the bioadhesive layer, and the outer surface of the balloon inhibits the adhesion of a bioadhesive layer to the balloon surface.

Another multilayer bioadhesive coating has been developed with the same bioadhesive polymer, containing an additional third protective layer impregnated with endothelial cell stimulant between the first and second coatings [200]. The coating layers are deposited in a discrete pattern by an aerosol jet application. The protective coating is weak and dissolves within a few minutes, and there is no interference between the bioadhesive coating and the tissue. Weber et al. developed a coating comprising a metal layer on the outer surface of the expandable membrane and a fibrous polymeric layer coated over the metallic coating, where the fibers were embedded in the pores of the polymeric layer [231]. The threads with a microsize diameter made up of polyamide or polyester facilitate a high amount of therapeutic agent loading. The polymeric fibers were coated with a surfactant to prevent entanglement between them. Simultaneously, the metallic coating on the balloon helped in the tracking of the device and provided a larger surface area of fibers for high drug loads. The therapeutic agent was released when the balloon membrane was inflated. Innovative coating methods make it possible to tune the amount of drugs available at the site.

The types of drug-coated balloons with regulatory approval status and clinical trials are presented in Table 14 [14].

## 8. Conclusions

Drug-eluting stents and drug-eluting balloons introduced in recent years for widespread use in CAD treatments inhibit the inflammatory process. They have antimigration and antiproliferative functions, as well as support tissue healing. Despite many clinical studies confirming the angiographic effectiveness of the new types of stents and balloons described here, there are still too-few long-term observations confirming the effectiveness of these treatment methods.

There are conflicting DES safety data on the frequency of late-stent thrombosis compared to bare metal stents [232,233,234]. Indeed, this is due to the different effectiveness and safety of individual types of DES, the number of which is systematically increasing [235]. Therefore, mere compliance with the Conformité Européenne (CE) directives without randomized clinical trial results does not guarantee comparable efficacy and safety for new types of DES [236].

The future of DES is not clear-cut and will depend primarily on technological advances, new structure design solutions, and an improved drug formula. An ideal stent should be characterized by flexibility, strength, and the construction optimally suited to the appropriate drug release [2]. One of the main directions of DES technology development is to modify their surfaces with improved polymer and nanocomposite materials. There is also undergoing work to improve the stent core using biodegradable and bioresorbable materials.

DES currently produced allow the use of different drugs and control the local sustainable release of active substances. Some metal stents have a special structure that enables direct coating without using polymer surface modifiers. Stents coated with biocompatible substances imitating tissue and cell surfaces are also available.

Much attention is paid to polymers as carriers of therapeutic agents. They are responsible for the mechanical maintenance of the drug, keeping the chemical stability of the drug, and regulating the drug-release profile. Until now, the properties of polymers did not meet all expected requirements of researchers. The durability of the coating, physical properties adapted to the vascular tissue, compliance with the nature of the drug, biocompatibility, and controlled and prolonged release of the drug are critical requirements for polymers used in stents. In practice, it is almost impossible for all the desired properties to be present in one polymer. The solution may be to use a polymer mixture.

Research is underway on new technologies to use biodegradable polymer-coated stents. The application of biodegradable polymers allows the use of large doses of the drug and reasonable remote control of the released treatment, as well as the complete degradation of the drug complex in a given time.

Recently, a polymer-free DES has been designed to make up for the increased risk of late-thrombosis and inflammation ascribed to polymers. The advanced polymer-free stent design uses porous surfaces and reservoirs to introduce significant amounts of anti-inflammatory and immunosuppressive drugs. Preclinical and clinical trials have reported the comparable performances of both polymer-free and polymer-coated stents [78]. However, some studies show that polymer-free stent technology offers long-term benefits and increased efficacy compared to traditional polymer-based DES [15].

In the past several years, the limitations of DES (late-thrombosis and inflammatory reactions) have induced research on other treatment alternatives for atherosclerosis. Thus, a nonstent technology in which the vessel wall receives the effective homogenous delivery of an antiproliferative drug from the surface of an inflated balloon was created. The treatment goal of enlarging the vessel lumen and local drug delivery was achieved without implanting a permanent object. Balloon catheters should have adequate flexibility, high mechanical strength, and be thinly walled. These physical features are enabled by the use of special core materials such as polyvinyl chloride, crosslinked polyethylene, polypropylene, polyamides, polyimides, polyesters like polyethylene terephthalate, polyethylene (naphthalene dicarboxylate), and nylon [194,195]. Among all active agents, paclitaxel and sirolimus are the preferred drugs to be applied to DEB [14]. There are different methodologies for loading the active substance onto the balloon, such as spraying, dipping, micro-pipetting, applying nanoparticles, and imprinting the medicine onto the rough surface of the balloon [221]. Researchers confirmed that excipients play an important role in the functionality of DEB. They are responsible for making the drug transfer to the arterial wall more effective. Currently, iopramide, urea, shellac, and butyryl-trihexyl citrate are the most commonly used excipients [213]. Controlling the release of drugs into the vessel wall, while inflating the balloon, is a vital issue in the DEB design process. The critical parameter of the safety and efficacy of the drug is that it should be released at a desirable rate and without a loss of effectiveness. Therefore, the limited sustainable release of the active substance with time passed after the use of a DEB is a major concern. Nevertheless, drug-eluting balloons, along with drug-eluting stents, are options in treating CAD. The supremacy of one of these solutions over the other in terms of short- and long-time effectiveness and post-procedure complications still needs more research.

Despite all issues discussed, drug-eluting stents and drug-eluting balloons are a significant alternative to “classical” cardiac surgery in the treatment of coronary artery disease.

## Figures and Tables

**Table 1:** Review of publications presenting the latest developments in drug-eluting stents (DES).

Authors	Year	Title	Abstract	Ref.
Chen et al.	2016	Coronary stent technology: a narrative review	A description of the evolution of coronary stent technology, the efficacy and safety of currently available devices, and the rationales for new-generation techniques in this domain.	[1]
Htay et al.	2005	Drug-Eluting Stent: A Review and Update	A summary of the recent development and progress of drug-eluting stents, followed by the results of their clinical trials.	[21]
Burt et al.	2006	Drug-eluting stents: A multidisciplinary success story	A comprehensive view of the disciplines related to the design and the development of drug-eluting stents, followed by a discussion on future directions in this domain.	[22]
Martin et al.	2011	Drug-eluting stents for coronary artery disease: A review	A review of both approved and most-promising proposals of drug-eluting stents. The study is a starting point for an indicator of the ways of the evolution of drug-eluting stents.	[23]
Doostzadeh et al.	2010	Recent progress in percutaneous coronary intervention: evolution of the drug-eluting stents focuses on the XIENCE V drug-eluting stent.	A discussion of clinical outcomes of drug-elution stents: clinical trials and development problems, design methods, and critical features, followed by an analysis of the future of this domain	[24]
Silber et al.	2008	Drug-eluting stents for diabetic patients. A critical appraisal of the currently available data from randomized trials	A review is summarizing the results of clinical trials and analysis for patients with coronary artery disease and parallel diabetosis.	[25]
Li et al.	2011	Recent developments in drug-eluting stents	A summary of recent developments of drug-eluting stents as a base for novel methods in the management of symptomatic coronary artery disease, followed by a discussion of problems associated with the usage of this technology.	[26]
Buchanan et al.	2017	Does the new generation of drug-eluting stents render bare-metal stents obsolete?	A review of the literature devoted to the safety and efficacy of drug-eluting stents and a comparison of this technique with bare-metal stents.	[27]
Fusaro et al.	2013	Drug-eluting stents for revascularization of infrapopliteal arteries: an updated meta-analysis of randomized trials	An updated meta-analysis of randomized trials investigating the outcomes of percutaneous revascularization with primary drug-eluting stenting in patients with atherosclerotic disease of infrapopliteal arteries.	[28]
Shlofmitz et al.	2019	Restenosis of Drug-Eluting Stents: A New Classification System Based on Disease Mechanism to Guide Treatment and State-of-the-Art Review	A new classification of in-stent restenosis by different mechanical, biological, and mixed etiologies, to enable individual treating of patients with drug-eluting stents to improve clinical outcome.	[29]
Wiesinger et al.	2019	Future developments in ureteral stents	A review of recent literature to summarize the most recent evidence on the use of ureteral stents, including the use of different materials and treatment of stent-related symptoms.	[30]
Lukman et al.	2019	Emerging of cardiovascular metal stent: A review on drug-eluting stent towards the utilization of herbal coating	A review of the utilization of various drugs as coating materials in identifying a possible alternative to overcome the current complications of DES. The discussion was divided into three sections: Stent; Commercial drug coating on DES; Herb coating on DES for cardiovascular application.	[31]
Wu et al.	2019	Polymer-free versus durable polymer drug-eluting stents in patients with coronary artery disease: A meta-analysis	A meta-analysis of randomized controlled trials to evaluate the safety and efficacy profiles of polymer-free drug-eluting stents compared with durable polymer drug-eluting stents.	[32]
Kommineni et al.	2018	Nonpolymer drug-eluting coronary stents	A review of nonpolymer drug-eluting stents loaded with different drugs like anti-inflammatory agents, antithrombotic, antiplatelet agents, immune suppressants, and others, followed by a description of surface modification techniques on stents like crystalline coating; microporous, macroporous, and nanoporous coatings; and chemically modified self-assembled monolayers.	[33]
Livingston et al.	2019	Coating Techniques and Release Kinetics of Drug-Eluting Stents	A review paper discusses recent drug-eluting stents designs utilizing individual or a combination of several coating techniques and their resulting drug-release profiles.	[34]

**Table 2:** Selected review publications on polymers applicable to DES.

Authors	Year	Title	Abstract	Ref.
Mori et al.	2017	Revisiting the role of durable polymers in cardiovascular devices	Presentation and discussion of the problems related to the 1st generation DP-DES, areas of success and failure of the 2nd generation DP-DES, as well as a summary of the advantages and disadvantages of BP-DES.	[17]
Rizas et al.	2016	Stent Polymers: Do They Make a Difference?	A review of various permanent (biostable) and biodegradable polymers (BPs) that are used on DES platforms, followed by a discussion of needed features: biocompatibility, lack of interaction with the active drug, appropriate drug-eluting kinetics, biological inertion after the drug has been wholly eluted, and mechanical stability.	[52]
Stewart et al.	2018	Implantable Polymeric Drug Delivery Devices: Classification, Manufacture, Materials, and Clinical Applications Implantable Polymeric Drug Delivery Devices: Classification, Manufacture, Materials, and Clinical Applications	A classification of the implantable drug delivery devices, as well as a description of the drug-release mechanisms, followed by a discussion on materials and manufacture methods, and finally, examples of clinical applications.	[53]
Strohbach et al.	2015	Polymers for Cardiovascular Stent Coatings. Review	Discussion on the parameters of tissue and blood cell functions to be considered to evaluate the biocompatibility of stent polymers, especially towards biodegradable polymers; additionally, a summary of the methods to assess these parameters in certain physiological conditions.	[54]
Joseph et al.	2018	Biomedical applications of polyurethane materials and coatings	A review summarizes state-of-the-art from 2014 to 2018 in the domain of polyurethane materials and coatings and their biomedical applications, taking into account the biocompatibility, biodegradability, and tailorable chemical and physical forms.	[55]
Englert et al.	2018	Pharmapolymers in the 21st century: Synthetic polymers in drug delivery applications Pharmapolymers in the 21st century: Synthetic polymers in drug delivery applications	A summary of the classes of synthetic polymers and their applications in polymer-drug conjugates, excipients, and in nano- and macroscopic drug carriers as coatings and as drugs.	[56]

**Table 3:** Drug-eluting stents (DES) with nonbiodegradable polymer surfaces available on the market or during clinical trials [58].

Trade Name.	Stent Platform	Polymer System	Drug	Drug Release (Days)	Manufacturer	Approval
Cypher^®^	SS	PEVA, PBMA, PCh	Sirolimus	40% (5) 85% (30) 100% (90)	Cordis Corporation (Hialeah, FL)	FDA, CE
Taxus^®^	SS	Poly(styrene-b-isobutylene-b-styrene)	Paclitaxel	<10% (28)	Boston Scientific (Marlborough, MA)	FDA, CE
Promus PREMIER^TM^	Pt-Cr	PBMA, poly(vinylidene-co-hexafluoropropylene)	Everolimus	71% (28) 100% (120)	Boston Scientific (Marlborough, MA)	FDA, CE
Xience V^®^	Co-Cr	PBMA, poly(vinylidene-co-hexafluoropropylene)	Everolimus	80% (28) 100% (120)	Abbot Vascular (Chicago, IL)	FDA, CE
Endeavor^®^	Co-Cr	Phosphorylcholine polymer	Zotarolimus	75% (2) 95% (15) 100% (28)	Metronic (Fridley, MN)	FDA, CE
Endeavor^®^ Resolute	Co-Cr	Blend of PVP, poly(hexyl methacrylate)-co-PVP-co-PVAc, and PBMA-co-PVAc (BioLinx)	Zotarolimus	50% (7) 70% (28) 100% (31)	Metronic (Fridley, MN)	FDA, CE
Firebird 2^®^	Co-Cr	Poly(styrene-butylene styrene)	Sirolimus	50% (7) 90% (30)	Essen Technology (Beijing, China)	Phase IV NCT01257373

Pt-Cr: platinum chromium, SS: stainless steel, Co-Cr: cobalt-chromium, PCh: phosphorylcholine polymer, PEVA: poly(ethylene-co-vinyl acetate), PBMA: poly(n-butyl methacrylate), and CE: Conformité Européenne.

**Table 4:** Drug-eluting stents (DES) with biodegradable polymers as coating materials [56].

Trade Name	Stent Platform	Polymer System	Drug	Drug Release (Days)	Manufacturer	Approval
Synergy^TM^	Pt-Cr	PLGA	Everolimus	(60) 50% (90) 100%	Boston Scientific (Marlborough, MA)	FDA, CE
Axxess^TM^	Nitinol	PLA	Biolimus A9	(30) 45%	Biosensors (Irvine, CA)	CE
BioMatrix Flex^TM^	SS	PLA	Biolimus A9	(30) 45%	Biosensors (Irvine, CA)	CE
Nobori^®^	SS	PLA	Biolimus A9	(30) 45%	Terumo (Somerset, NJ)	CE
Supralimus^®^	SS	PLLA-PLGA-PCL-PVP	Sirolimus	(48) 100%	SMT (Mumbai, India)	CE
Orsiro	Co-Cr	PLLA + silicon carbide	Sirolimus	(30) 50% (90) 80%	Biotronik (Poznań, Poland)	CE
BioMime^TM^	Co-Cr	PLLA + PLGA PLLA + PLGA	Sirolimus	(30) 100%	Meril (Gujarat, India)	CE
Inspiron^®^	Co-Cr	PLLA, PDLLGA PLLA, PDLLGA	Sirolimus	(10) 60% (45) 100%	SciTech Medical (Aparecida de Goiânia, Brasil)	Phase IV NCT01856088
Firehawk^®^	Co-Cr	PDLLA	Sirolimus	(90) 90%	MicroPort Medica (Shanghai, China)	CE
DESyne^®^ BD	Co-Cr	PLA	Novolimus M	(90) 90%	Elixir^®^ (Milpitas, CA)	CE
MiStent SES^®^	Co-Cr	PLGA	Sirolimus	(270) 100%	Micell Technologies (Durham, SC)	CE
Tivoli^®^	Co-Cr	PLGA	Sirolimus	(7) 50% (28) 80%	Essen Technology (Beijing, China)	Phase III NCT02448524

Pt-Cr: platinum chromium, SS: stainless steel, and Co-Cr: cobalt-chromium.

**Table 5:** Biodegradable polymers.

Structure	Products of Degradation	
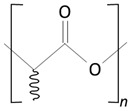	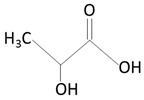	
polylactic acid (PLA)	lactic acid (LA)	
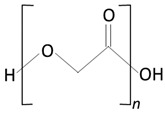	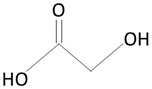	
polyglycolic acid (PGA)	glycolic acid (GA)	
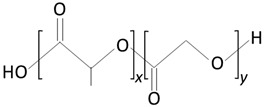	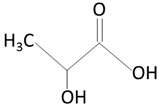	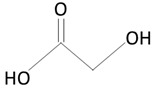
poly(lactic-co-glycolic acid) (PLGA)	lactic acid (LA)	glycolic acid (GA)
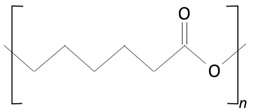	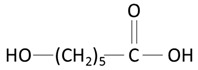	
poly(caprolactone) (PCL)	caproic acid	
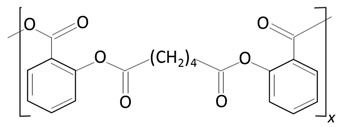	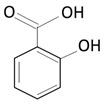	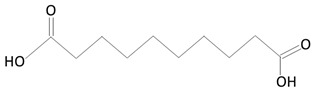
poly(anhydride ester)	salicylic acid (SA)	sebacic acid

**Table 6:** Mechanical and thermal properties of the most commonly used medical biodegradable polymers.

Material	E	*σ*	*ε*	T_g_	T_melt_	Loss of Mech. Prop.	Total Degradation
	(GPa)	(MPa)	(%)	(°C)	(°C)	(Months)	(Months)
PLLA	3.4–4.8	10–100	2–6	60–65	170–180	6	24–67
PGA	6.8–12.5	70–647	min	35–40	180–230	1–2	6–12
PLGA (D/L/PLG) 85/15-50/50	2	20–50	3–10	45–55	-	1–4	2–6
PCL	0.3–0.4	16–23	300–700	−60	59–64	0.8	>34

T_g_: Glass transition temperature and T_melt_: melting point.

**Table 7:** Drug-eluting stents (DES) with fully biodegradable scaffolds and coatings.

Trade Name	Stent Platform	Polymer System	Drug	Drug Release (DAYS)	Manufacturer	APPROVAL
Absorb^TM^	PLLA	PDLLA	Evorolimus	(28) 80%	Abbot Vascular (Northbrook, IL)	FDA approval
DESolve^®^	PLLA	PLLA	Novolimus^TM^	(180–270) 100%	Elixir^®^ (Milpitas, CA)	CE approval
Dreams I	Mg	PLGA	Paclitaxel	(90) 100%	Biotronik (Poznań, Poland)	Phase 0 NCT01168830
Dreams II	Mg	PLLA	Sirolimus	n.a.	Biotronik (Poznań, Poland)	Phase 0 NCT01960504
ReZolve2	PTD-PC	n.a.	Sirolimus	(90)~100%	REVA (San Diego, CA)	Clinical study NCT01845311

Mg: magnesium, PTD-PC: poly-tyrosine-derived polycarbonate, n.a.: not applicable.

**Table 8:** Agents used in drug-eluting stents [21].

Antineoplastics and Anti-Inflammatory Immunomodulators	Antiproliferative	Migration Inhibitors and ECM Modulators	Enhanced Healing and Re-Endothelialization Factors
Sirolimus	QP-2, Taxol (Paclitaxel)	Batimastat	BCP671
Tacrolimus	Actinomycin	Prolyl hydroxylase inhibitors	VEGF
Everolimus	Methotrexate	Halofuginone	Estradiols
Leflunomide	Angiopeptin	C-proteinase inhibitors	NO donor compounds
M-Prednisolone	Vincristine	Probucol	EPC antibodies
Dexamethasone	Mitomycin		Biorest
Interferon r-1b	Statins		
Mycophenolic acid	C-myc antisense		
Mizoribine	Abbott ABT-578		
Cyclosporine	Resten ASE		
Tranilast	2-chloro-deoxyadenosine PCNA ribozyme		

ECM: extracellular matrix, EPC: endothelial progenitor cells, NO: nitric oxide, PCNA: proliferating cell nuclear antigen, VEGF: vascular endothelial growth factor, and QP-2: 7-hexanoyltaxol.

**Table 9:** Clinical trials using agents excluding sirolimus and paclitaxel [21].

Tacrolimus	Present I–III	Preliminary Safety Evaluation of Nanoporous Tacrolimus-Eluting Stents
	EVIDENT	The endovascular investigation determining the safety of new tacrolimus-eluting stent grafts.
Everolimus	FUTURE I–IV	First used to underscore the reduction in restenosis with everolimus.
	SPIRITS-FIRST	
M-Prednisolone	IMPRESS	Immunosuppressive therapy for the prevention of restenosis after coronary artery stent implantation.
Dexamethasone	STRIDE	The study of antirestenosis with a BiodivYsio dexamethasone-eluting stent.
	EMPEROR	Evaluation of the 9α-F-16 methylprednisolone (dexamethasone)-eluting stent on the reduction of restenosis.
	DESIRE	Dexamethasone-eluting stent, Italian registry.
	SAFE	Sorin and aspirin following elective stenting.
Mycophenolic acid	IMPACT	Inhibition with MPA of a coronary restenosis trial.
Batimastat	BATMAN	BiodivYsio batimastat SV stent versus balloon angioplasty for the reduction of restenosis in small coronary arteries.
	BRILLIANT	Batimastat (BB-94) antirestenosis trial utilizing the BiodivYsio local drug delivery PC stent.
Actinomycin	ACTION	Recruitment in the actinomycin-eluting stent improves outcomes by reducing neointimal hyperplasia.
Angiopeptin	SWAN	Stent with angiopeptin.
Medtronic ABT-578	ENDEAVOR I–III	A randomized controlled trial to evaluate the safety and efficacy of the Medtronic AVE ABT-578- eluting driver^TM^ coronary stent in de novo native coronary artery lesions
Abbott ABT-578	Zomaxx 1	Zomaxx coronary drug-eluting stent for de novo lesion in coronary arteries.
Estradiols	EASTER	Estrogen and stent to eliminate restenosis.
NO donor compounds	NOBLESSE	Nitric oxide through a biodegradable layer elective study for safety and efficacy.
EPC antibodies	HEALING I–II	Healthy endothelial accelerated lining inhibits neointimal growth.

MPA: mycophenolic acid, PC: phosphorylcholine, and SV: small vessel.

**Table 10:** Review of publications in which the authors discussed various aspects of drug-eluting balloons (DEBs) in the last five years.

Authors	Year	Title	Abstract	Ref
Borhani et al.	2018	Cardiovascular stents: overview, evolution, and next generation. Review	A discussion on different techniques for stent design, mainly based on recent advances in drug-eluting stents.	[2]
Bukka et al.	2018	Drug-eluting balloon: design, technology and clinical aspects. Topical review	A review and discussion of the evolution, rationale, and comparison of the drug-eluting balloons currently available on the market, with a comparison of different coating techniques.	[14]
Naghi et al.	2016	New developments in the clinical use of drug-coated balloon catheters in peripheral arterial disease	A review summarizes currently available clinical data on the application of drug-coated balloons, followed by a presentation of new paclitaxel drug-coated balloons.	[173]
Byrne et al.	2013	Drug-coated balloon therapy in coronary and peripheral artery disease Review	A review of the clinical applications of balloons coated with drugs in the treatment of coronary and peripheral artery disease.	[174]
Schorn et al.	2017	The LUTONIX^®^ drug-coated balloon: A novel drug delivery technology for the treatment of vascular disease	A review summarizes the development of the LUTONIX^®^ drug-coated balloon catheter.	[175]
Cortesea et al.	2012	A comprehensive review of preclinical and clinical data	A review of specific parameters of the paclitaxel-coated balloons for the treatment of coronary artery disease.	[176]
Loh et al.	2012	Paclitaxel Drug-Coated Balloons A Review of Current Status and Emerging Applications Native Coronary Artery De Novo Lesions	A review of the role of drug-coated balloon DCB in de novo coronary lesions based on clinical evidence.	[177]
Katsanos et al.	2016	Comparative Effectiveness of Plain Balloon Angioplasty, Bare Metal Stents, Drug-Coated Balloons, and Drug-Eluting Stents for the Treatment of Infrapopliteal Artery Disease: Systematic Review and Bayesian Network Meta-analysis of Randomized Controlled Trials.	A meta-analysis of randomized controlled trials comparing bare-metal stents, paclitaxel-coated balloons, and drug-eluting stents with balloon angioplasty or with each other in the infrapopliteal arteries.	[178]
Zhang et al.	2017	Systematic Review and Meta-Analysis of Drug-Eluting Balloon and Stent for Infrapopliteal Artery Revascularization.	A review and meta-analysis of the current available studies investigating outcomes of drug-eluting balloons and drug-eluting stents in the treatment of infrapopliteal artery disease.	[179]
Katsanos et al.	2014	Bayesian network meta-analysis of nitinol stents, covered stents, drug-eluting stents, and drug-coated balloons in the femoropopliteal artery.	A meta-analysis of randomized controlled trials comparing bare nitinol stents, covered nitinol stents, paclitaxel- or sirolimus-eluting stents, and paclitaxel-coated balloons with plain balloon angioplasty or with each other.	[180]
Spiliopoulos et al.	2019	Current evidence of drug-elution therapy for infrapopliteal arterial disease.	A review summarizes and discussing data related to the application of infrapopliteal drug-elution devices and their future perspectives.	[181]
Chen et al.	2018	Drug-delivering endovascular treatment versus angioplasty in artery occlusion diseases: a systematic review and meta-analysis.	A comparison of the efficacy of drug-coated balloons and drug-eluting stents with percutaneous transluminal angioplasty in patients with femoropopliteal or infrapopliteal arterial occlusive disease.	[182]
Wua et al.	2019	Is There a Safety Concern for Drug-Coated Balloons in Peripheral Arterial Disease?	A description of the evolution of endovascular therapy for peripheral arterial disease, with highlights regarding the recent debates on the long-term safety of the drug-coated devices for the treatment of this disease.	[32]
Caradu et al.	2019	Systematic Review and updated meta-analysis of the use of drug-coated balloon angioplasty versus plain old balloon angioplasty for femoropopliteal arterial disease	A review of the use of drug-coated balloons in the management of femoropopliteal disease and a comparison of this technique with plain old balloon angioplasty.	[183]
Yang et al.	2019	A meta-analysis of the effects of drug-coated balloons among patients with small-vessel coronary artery disease	Clinical evaluation of drug-coated balloons for patients with small-vessel coronary artery disease.	[184]
Li et al.	2019	Drug-coated balloon versus drug-eluting stent in de novo small coronary vessel disease A systematic review and meta-analysis	A discussion on the safety and efficacy of the drug-coated balloons and the drug-eluting stents.	[185]
Kayssi et al.	2019	Drug-eluting balloon angioplasty versus uncoated balloon angioplasty for the treatment of in-stent restenosis of the femoropopliteal arteries	A description of the role of drug-eluting technologies, such as drug-eluting balloons, in the management of in-stent restenosis, summarized by the analysis of the efficacy of drug-eluting balloons compared with conventional uncoated balloon angioplasty in people with in-stent restenosis of the femoropopliteal arteries.	[186]
Lindquist et al.	2018	Drug-Eluting Balloons and Drug-Eluting Stents in the Treatment of Peripheral Vascular Disease	A review of the usage of drug-eluting technologies in the applications addressing the peripheral arterial system.	[187]
Mohiaddin et al.	2018	Drug-Coated Balloon-Only Percutaneous Coronary Intervention for the Treatment of De Novo Coronary Artery Disease: A Systematic Review	A review of stentless a drug-coated-balloon-only angioplasty in de novo coronary artery disease, comparing more than 40 studies examining the effects of drug-coated-balloon-only percutaneous coronary intervention in a variety of clinical scenarios, including small vessels, bifurcations, calcified lesions, and primary percutaneous coronary intervention.	[188]
Liuet al.	2018	Treatment of Drug-Eluting Stent In-Stent Restenosis With Drug-Eluting Balloons: A Systematic Review and Meta-Analysis	A description of the application of drug-eluting stents in the treatment of in-stent restenosis, with the outcome of investigating the death, myocardial infarction, and target-lesion revascularization at longest available follow-up (up to 3 years).	[189]
Meneguz-Moreno et al.	2018	Drug-Coated Balloons: Hope or Hot Air: Update on the Role of Coronary DCB	A review of applications of drug-coated balloons with the clinical evidence, to enumerate profits, especially in the implementation of bare-metal stents and drug-eluting stents in-stent restenosis.	[190]
Kokkinidis et al.	2018	Second-generation drug-eluting stents versus drug-coated balloons for the treatment of coronary in-stent restenosis: A systematic review and meta-analysis	A comparison of second-generation drug-eluting stents and drug-coated balloons for the treatment of coronary artery in-stent restenosis.	[191]
Merinopouloset al.	2018	Percutaneous Coronary Intervention in the Elderly: Are Drug-coated Balloons the Future?	A review and history of percutaneous coronary intervention and the application of drug-coated balloons, especially within the elderly population.	[192]

**Table 11:** Types of polymeric materials and characteristics for balloon fabrication [14].

Material	Tensile Stress	Compliance	Maximum Sustainable Pressure
	(10^4^ psi)	(%)	(atm)
Polyethylene terephthalate (PET)	3–6	Low compliance (0–10)	18–27
Nylon	2–4	Semi-compliant (5–15)	5–18
Polyethylene with additives	1	Compliant (>15)	10
Polyvinyl chloride (PVC)	<1	Compliant (>15)	6–8
Polyurethane used along with nylon	1–2	Semi-compliant (5–15)	10

**Table 12:** Types of therapeutic agents used in drug-eluting balloons [14].

Class of Therapeutic Agent	Examples	Mechanism of Action
Antiplatelet	Aspirin, clopidogrel	Reduces blood clotting
Anti-inflammatory	Glucocorticoids, betamethasone, dexamethasone prednisolone	Inhibits monocyte and macrophage function and influences smooth muscle cell proliferation
Antihyperlipidemic	Statins (simvastatin, pravastatin), probucol	Decreases blood cholesterol level
Antiproliferative	Taxanes (paclitaxel docetaxel) limus (sirolimus, everolimus, tacrolimus)	Inhibits the G1 or G2 phase and the proliferation of cells
Cytotoxic antibiotics	Actinomycin-D	Inhibits the G1 phase and the proliferation of cells
Antithrombogenic agents	Heparin, urokinase	Prevents the formation of thrombin

**Table 13:** Excipients used in a drug-eluting balloon [14].

	Excipient	Role	Ref.
Bioadhesives	Amino acids (DOPA)	To deliver the therapeutic agent from the vehicle and gain adhesion to the vessel	[142,146]
	Adhesive surface proteins (microbial surface components recognizing adhesive matrix molecules)	To adhere to the lesion site, produced by pathogens	
	Polymer materials (polysaccharides, alginic acid, PVA, etc.)	To deliver the therapeutic agent from the vehicle and gain adhesion to the vessel	
	Minigel particles (poly(N-iso propyl acrylamide))	To gain adhesion to the vessel at body temperature and behave as a liquid	
	Endothelial cell stimulant (mono- and disaccharides and polymers)	Promotes the uptake of a therapeutic agent when it comes into contact with endothelial cells lining the vasculature	
Hydrophilic carriers	Shellac	To increase the adhesion of paclitaxel, considered as a superior excipient in binding paclitaxel	[146,218]
	Urea	To increase homogenous delivery of a drug	[146]
	Iopramide	To monitor balloon movements, a radio spacer agent used in angiography	[146,148]
	Butyryl-trihexyl citrate	Hydrophilic carrier in binding a lipophilic drug to the vessel wall	[146]
Lipophilic lubricant	C6-C30 magnesium, zinc, calcium, or ammonium Monocarboxylic acid salt, talc, or magnesium stearate	To decrease the frictional force between the polymer layer and balloon and to increase drug binding to the balloon	[150]
Antioxidants	Butylated hydroxyl toluene	To prevent the decomposition of the drug through oxidation and promote adherence of medicine to the balloon	[219]

**Table 14:** Types of drug-coated balloons with their regulatory approval status and clinical trials. BMS: bare-metal stents and ISR: in-stent restenosis.

Balloon	Application	Clinical trials	Groups	CE Approval	FDA Approval
PACCOCATH^®^ COTAVANCE^TM^ MEDRADIN, USA	Infrainguinal and iliac arteries	ISR I	PACCOCATH^®^ DEB versus uncoated DEB	Yes, 2011	No
		ISR II (12 months)			
		FEMPAC	Uncoated balloon, DEB		
		THUNDER	Plain balloon, DEB, a drug with contrast media		
DIOR^®^, Eurocor GmbH, Germany	Coronary arteries	DIOR Registry	DEB after BMS-ISR, DES-ISR	No	No
	Small coronary arteries	DEBUIT	DEB after BMS, DEB	YES, 2007 for coating	
		PICCOLETO	DES (versus) DEB		
		VALENTINES II	Lesions with DEB		
		DEB-AMI	DEB+BMS, BMS, Taxus^®^ DES		
IN.PACT^™^Admiral^™^, Medtronic, USA	Peripheral artery diseases (upper leg)	IN.PACT^™^ SFA I	DEB (versus) plain balloon	Yes, 2009	PMA 2015
SeQuent^®^ Please, B. Braun Vascular Systems, Germany	Coronary artery and small vessels	PEPCAD I	Sequent^™^Please (versus) Sequent^™^Please+BMS	Yes, 2009	No
		PEPCAD II	Sequent™Please (versus) Taxus^®^		
		PEPCAD III	Sequent^™^Please + Coroflex^™^ DEBlue (versus) Cypher^®^		
		PEPCAD IV	Sequent^™^Please (versus) Coroflex^™^DEBlue and Taxus^®^		
		PEPCAD V	Sequent^™^Please (versus) Coroflex^™^DEBlue and Taxus		
		PEPCAD DES	Sequent^™^Please (versus) plain balloon		
		PEPCADCTO	Sequent^™^Please+BMS (versus) Taxus^®^		
		INDICOR	BMS after Sequent^™^Please (versus) Sequent^™^Please after BMS		
		PERFECT	Sequent^™^Please + EPC stent (versus) EPC stent		
		ISAR DESIRE	Sequent^™^Please, DES, uncoated balloon		

## Abbreviations

BCS	Bioresorbable Cardiovascular Scaffold
BMS	Bare Metal Stent
CAD	Coronary Artery Disease
CDDS	Controlled Drug Delivery System
DDS	Controlled Drug Delivery Systems
DEB	Drug-Eluting Balloon
DCB	Drug-Coated Balloon
DES	Drug-Eluting Stent (Drug-Coated Stent)
ISR	In-Stent Restenosis
PCS	Polymer Coating Stents
PDLLA	Poly (D, L-Lactide)
PLLA	Poly (L-Lactic Acid)
PTCA	Percutaneous Trans Luminal Coronary Angioblasts)
ST	Stent Thrombosis
SMP	Shape-Memory Polymer
